# Interfacial H relay and site regeneration in Mn single atom-Ru cluster hybrids for efficient alkaline HER

**DOI:** 10.1039/d6sc06709c

**Published:** 2026-11-04

**Authors:** Shangyan Zhou, Fei Liu, Qiandan Ren, Huarui Wang, Yanyu Zhang, Quande Man, Ju Guo

**Affiliations:** a Department of Chemical Engineering, School of Chemistry and Chemical Engineering, Guizhou University Guiyang Guizhou 550025 China ce.feiliu@gzu.edu.cn; b Key Laboratory of Carbon-based Energy Molecular Chemical Utilization Technology in Guizhou Province, Guizhou University Guiyang Guizhou 550025 China; c Guizhou Provincial Key Laboratory of Green Catalysis and Materials for Resource Conversion, Guizhou University Guiyang Guizhou 550025 China; d Guizhou Provincial Technological Innovation Centre of Resource Coupling and Deep Processing for Sulfur-Phosphorus-Titanate-Iron, Guizhou University Guiyang Guizhou 550025 China; e School of Brewing Engineering, Moutai Institute Renhuai Guizhou 564507 China

## Abstract

It is widely recognized that constructing synergistic catalysts between single atoms and clusters is a potential strategy to enhance the kinetics of the alkaline hydrogen evolution reaction (HER). However, the unclear reaction mechanism hinders the understanding of synergy and complicates the rational design. Herein, we report a hybrid electrocatalyst with Mn single atoms and Ru clusters co-anchored on N-doped carbon (Ru_AC_/Mn_SA_NC), which enables an interfacial H relay for accelerated HER. *In situ* Raman spectroscopy and DFT calculations collectively demonstrate that the oxophilic Mn sites readily dissociate water, and the resulting H species are relayed to adjacent Ru nanoclusters, which possess a near-optimal Δ*G*_H*_. This interfacial H* relay enriches reactive H* on Ru while regenerating the Mn sites for subsequent water activation, thereby enabling continuous catalytic turnover through Mn-site regeneration. Moreover, the Mn single atom alters the electronic configuration of Ru and weakens the excessive Ru–H interaction, thereby facilitating more efficient H* transfer and H_2_ desorption. As a result, the Ru_AC_/Mn_SA_NC catalyst exhibits an exceptionally low overpotential of 66 mV at 50 mA cm^−2^ under alkaline conditions and excellent long-term durability exceeding 600 hours. This work not only presents a high-performance catalyst but also provides profound mechanistic insights into the design of cooperative catalytic systems for energy conversion.

## Abstract 2

In this work, Mn single atoms promote water dissociation and relay hydrogen to adjacent Ru clusters for alkaline HER. The catalyst achieves 50 mA cm^−2^ at 66 mV overpotential with stability exceeding 600 h.

## Graphical abstract

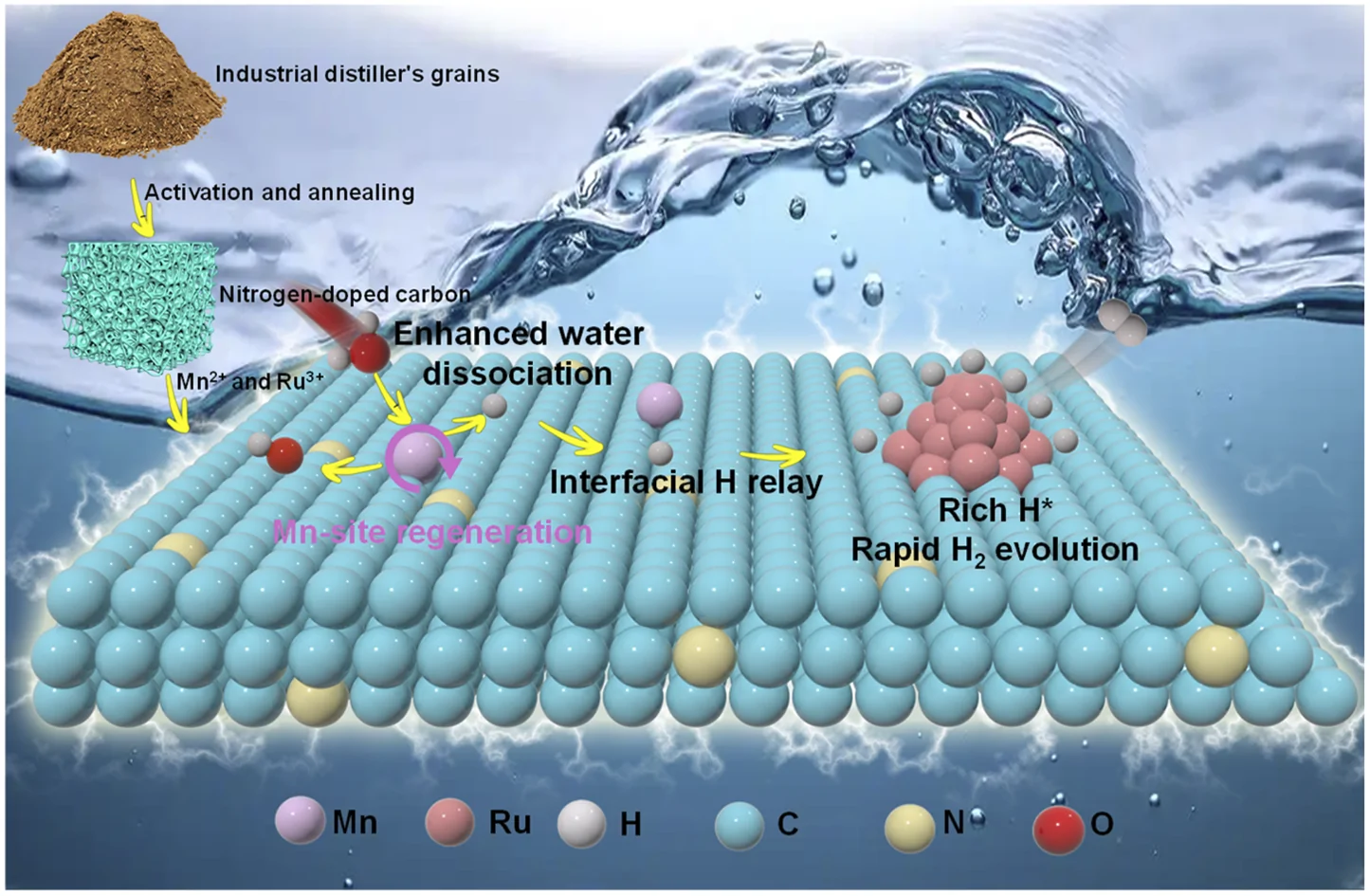

## 1. Introduction

Hydrogen energy, owing to its clean combustion, high energy density, and suitability for large-scale energy storage, is considered a promising alternative to traditional fossil fuels.^1–4^ Water electrolysis is a promising method for hydrogen production.^5,6^ However, the alkaline hydrogen evolution reaction (HER) at the cathode involves an additional water-dissociation step, which requires extra energy and consequently leads to sluggish reaction kinetics and a higher overpotential.^7–9^ Previous studies have demonstrated that water activation and HER kinetics can be strongly influenced by the local chemical environment of the active sites and, in some catalyst systems, by electrochemically induced surface reconstruction under alkaline or neutral conditions.^10,11^ Meanwhile, high-current-density operation and mass-normalized activity are important complementary metrics for evaluating the practical potential and intrinsic utilization efficiency of HER catalysts. Therefore, it is important to reduce the energy barrier for water dissociation, achieve suitable adsorption of reaction intermediates, and rationally regulate the local environment of active sites under alkaline conditions.

Research has shown that transition metals (Fe, Co, Ni, Mo, and Ru) are ideal substitutes for Pt-based electrocatalysts due to their enhanced adsorption affinity. Ruthenium (Ru) has garnered significant attention as a promising alternative to platinum due to its favorable hydrogen binding energy.^12–16^ However, Ru-based catalysts still face intrinsic limitations, including strong hydrogen adsorption, weak hydrolysis ionization energy and a tendency toward nanoparticle aggregation, which restrict their catalytic efficiency and long-term stability.^17,18^ To address these issues, recent studies have explored the integration of Ru with oxophilic single-atom sites to tune the local electronic environment and enhance the alkaline HER kinetics through synergistic effects. For instance, Fe-doped Ru nanoparticles (RuFe/FeNC) have been shown to optimize the electrode–electrolyte interface by enriching H-down water molecules and facilitating water dissociation.^19^ Similarly, the coupling of Ru clusters with Y single atoms (Ru-YNC) has been reported to trigger a cascade electrocatalysis mechanism, decoupling water dissociation and hydrogen evolution steps.^20^ These studies highlight the importance of spatially coupling complementary active sites, yet the pathways by which reaction intermediates are transferred between these sites remain insufficiently understood.

Herein, we report a highly efficient and stable HER catalyst comprising Mn single atoms and Ru nanoclusters co-anchored on N-doped carbon (Ru_AC_/Mn_SA_NC). Mn single atoms, owing to their oxophilicity, act as primary centers for water adsorption and dissociation, while the adjacent Ru nanoclusters function as highly active centers for hydrogen adsorption and recombination. The spatial proximity of Mn single atoms and Ru nanoclusters enables functional division between water activation and hydrogen evolution, while facilitating interfacial transfer of H intermediates between the two sites. Moreover, the introduction of Mn not only promotes water activation but also refines the size of Ru nanoclusters, enhancing the exposure of active sites and strengthening metal–support interactions. As a result, the Ru_AC_/Mn_SA_NC catalyst exhibits exceptional HER performance in alkaline media, achieving an ultralow overpotential of only 66 mV at 50 mA cm^−2^ and remarkable stability over 600 hours—surpassing most state-of-the-art Ru-based catalysts. This work offers a new strategy for designing high-performance HER electrocatalysts *via* atomic-level integration of complementary metal sites.

## 2. Materials and methods

### 2.1 Materials

Industrial distiller's grains were collected from a distillery in Guizhou, China. Potassium hydroxide (KOH, 95 wt%), hydrochloric acid (HCl, 36–38 vol%), manganese acetate [Mn(CH_3_COO)_2_, 99 wt%], and ruthenium chloride (RuCl_3_, 45–55 wt%) were obtained from Macklin Biochemical Co., Ltd. All chemicals were directly used as received without additional purification.

### 2.2 Synthesis of biomass carbon

Industrial distiller's grains were combined with KOH at a mass ratio of 1 : 2 and dispersed in 150 mL of deionized water. The suspension was maintained under continuous stirring at room temperature for 24 h, followed by drying at 120 °C for 12 h. The dried precursor was subsequently pyrolyzed at 800 °C for 2 h under an N_2_ atmosphere, using a heating rate of 5 °C min^−1^. After cooling, the carbonized product was treated with 0.5 M HCl to remove residual KOH and other soluble species, and the resulting solid was collected as the nitrogen-doped carbon (NC) support.

### 2.3 Synthesis of Ru_AC_/Mn_SA_NC

Nitrogen-doped carbon (NC) powder (0.1 g) was dispersed in 20 mL of a mixed solvent (deionized water/ethanol = 1 : 1 by volume) and sonicated for 30 min to achieve a homogeneous suspension. Then, 2 mL of 0.5 M manganese acetate solution was added dropwise to the NC dispersion under continuous stirring, and the mixture was stirred for 12 h to facilitate the adsorption of Mn^2+^ ions onto the NC support. Thereafter, the resulting solid was collected, dried, and finally calcined at 500 °C for 2 h in an N_2_ atmosphere to obtain the Mn_SA_NC catalyst. Subsequently, 10 mg of the ruthenium precursor (RuCl_3_) was introduced into the Mn_SA_NC containing 10 mL of aqueous solution, followed by further stirring for 12 h to ensure complete co-loading of both metal species. The slurry was subsequently subjected to drying in a vacuum oven at 60 °C, yielding a powdered precursor. The dried powder was annealed in a tube furnace under a 5% H_2_/95 N_2_ atmosphere. The temperature was raised to 800 °C at a heating rate of 5 °C min^−1^ and maintained for 2 h. The pyrolyzed product was then washed with 0.5 M H_2_SO_4_ to remove unstable and unanchored metal species. Finally, the sample was centrifuged, rinsed repeatedly with deionized water until neutral pH was achieved, and then dried to obtain the final catalyst. Ru_AC_/Mn_SA_NC-without KOH has the same Mn/Ru components but was synthesized without KOH activation, and Ru_AC_/NC was synthesized at 500 °C and contains Ru clusters supported on KOH-activated N-doped carbon but lacks Mn–N_4_ sites.

## 3. Results and discussion

Ru_AC_/Mn_SA_NC was synthesized *via* an immersion–reduction approach (Scheme 1). For comparison, Ru_NPs_/NC and Mn_SA_NC were prepared similarly but without Mn precursor addition and Ru precursor addition, respectively. Scanning electron microscopy (SEM) images reveal the rich porous structures for nitrogen-doped carbon (NC) with KOH activation (Fig. S1a) relative to nitrogen-doped carbon (NC) without KOH activation (Fig. S1b). This pore structure can enhance mass transport of reactants (H_2_O/OH^−^) and evolved H_2_ gas, while simultaneously increasing accessible active sites and optimizing triple-phase boundaries for improved HER kinetics. In addition, transmission electron microscopy (TEM) analysis was also conducted to examine the morphology and structure of the as-prepared catalysts.^21^ As exhibited in Fig. S2a, the MnNC catalyst does not show nanoparticles (NPs) on the NC substrate, preliminarily indicating that the Mn species in the MnNC catalyst are present as single atoms. The atomic dispersion of Mn species is unambiguously demonstrated by aberration-corrected high angle annular dark-field scanning transmission electron microscopy (HAADF-STEM), wherein only numerous isolated bright dots, corresponding to individual Mn atoms, are visible against the NC support. The TEM image of Ru_NP_/NC exhibits some NPs with an average particle size of 4.67 nm on the carbon support, indicating that Ru^3+^ has been successfully reduced to Ru NPs (Fig. S3a). Elemental mapping of Ru_NP_/NC revealed a homogeneous distribution of N, C, and O, whereas the Ru signal was exclusively localized at the nanoparticle sites, directly visualizing the successful formation of Ru NPs (Fig. S3b). Similarly, Ru_AC_/Mn_SA_NC exhibits a significant number of particles, featuring an average particle size of 1.33 nm and displaying a *d*-spacing of 0.219 nm, which align closely with the specific crystal planes of Ru (101), proving the successful synthesis of the Ru atomic cluster (AC) (Fig. 1a and b). Significantly, Mn species are not observed in these images, indicating that they are present as isolated atoms within the Ru_AC_/Mn_SA_NC structure. As presented in Fig. 1c, isolated bright spots (red circles) in Ru_AC_/Mn_SA_NC provide strong evidence for the existence of single Mn atoms. Comparing the particle sizes of Ru_AC_/Mn_SA_NC and Ru_NP_/NC, it is found that Ru_AC_/Mn_SA_NC is much smaller than Ru_NP_/NC, which is attributed to the presence of Mn inhibiting the aggregation of Ru atomic clusters, consequently increasing the accessibility of active sites and reinforcing the interaction between the metal and the support. Elemental mapping analysis of Ru_AC_/Mn_SA_NC (Fig. 1d) distinctly reveals a uniform distribution of Ru, Mn, C, and N throughout the sample. Besides, the inductively coupled plasma mass spectrometry (ICP-MS) analysis shows that the content of Ru and Mn in Ru_AC_/Mn_SA_NC is 9.87 wt% and 1.04 wt%, respectively (Table S1).

**Scheme 1:**
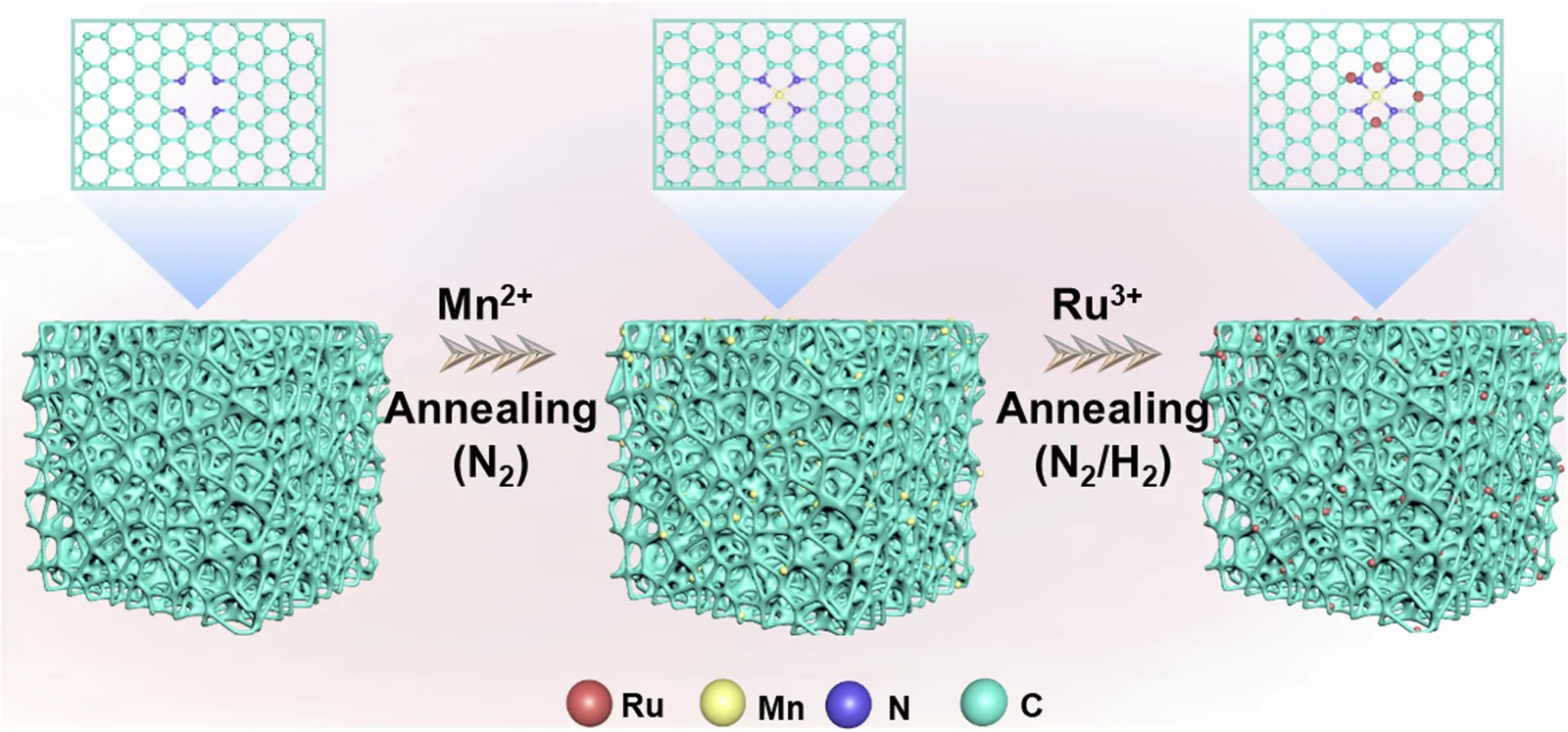
Schematic illustration of the Ru_AC_/Mn_SA_NC catalyst synthesis process.

**Fig. 1:**
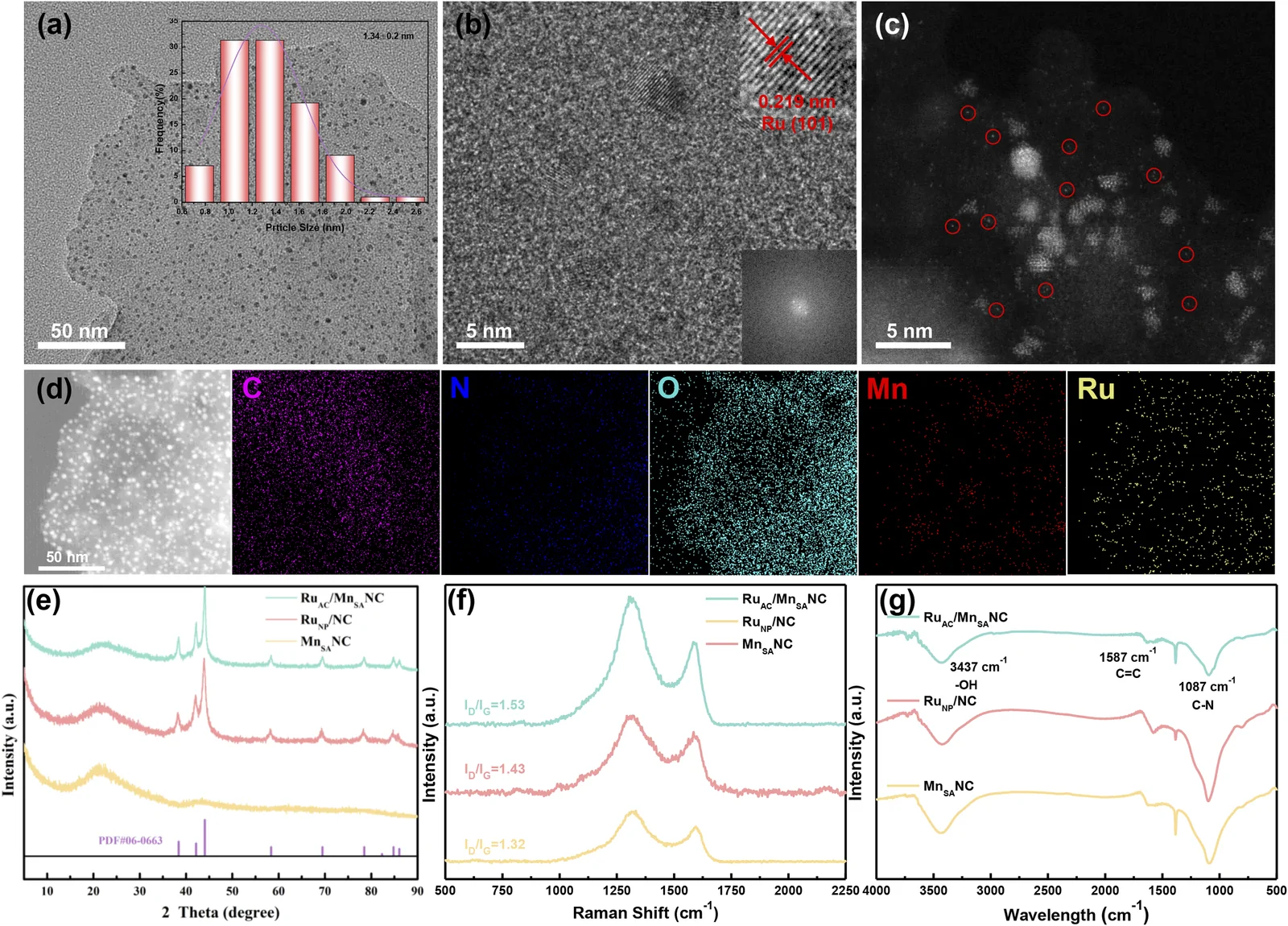
(a) TEM and (b) HRTEM images of Ru_AC_/Mn_SA_NC. (c) Aberration-corrected HAADF STEM image of Ru_AC_/Mn_SA_NC. (d) HAADF-STEM image and corresponding EDS spectroscopy elemental mapping of Ru_AC_/Mn_SA_NC. (e) XRD patterns, (f) Raman spectra and (g) FTIR spectra of Ru_AC_/Mn_SA_NC, Ru_NP_/NC, and Mn_SA_NC.

X-ray diffraction (XRD) was performed to characterize the crystal structures of the as-prepared catalysts. As presented in Fig. 1e, no diffraction peaks can be observed in the Mn_SA_NC catalyst, indicating that Mn exists in the form of a single atom, which aligns with the results of TEM and HAADF-STEM. However, Ru_AC_/Mn_SA_NC and Ru_NP_/NC exhibit the diffraction peak of Ru (PDF#06-0663), further implying the existence of Ru AC and Ru NP in Ru_AC_/Mn_SA_NC and Ru_NP_/NC, respectively. Moreover, no Mn-related crystalline phases are detected, further confirming the atomic dispersion of Mn.^22^ More importantly, the characteristic Ru (101) peak at ∼43.8° (PDF#06-0663) shifts to higher angles in Ru_AC_/Mn_SA_NC compared to Ru_NP_/NC, suggesting lattice contraction of Ru nanoparticles due to electronic interactions with adjacent Mn single atoms (Fig. S4a).^23^ Additionally, the attenuation of the broad C(002) peak at ∼21.5° in Ru_AC_/Mn_SA_NC indicates that dual-metal incorporation induces more pronounced structural disorder and defects than its single-metal analogue, thereby facilitating the HER process (Fig. S4b).^24^ As revealed in Fig. 1f, the Raman spectrum of Ru_AC_/Mn_SA_NC exhibits a pronounced increase in the *I*_D_/*I*_G_ ratio (1.53) compared to those of Ru_NPs_/NC (1.43) and Mn_SA_NC (1.32), indicating enhanced defect density induced, which is consistent with the result of XRD.^25^ As shown in Fig. 1g, the FTIR spectra reveal several characteristic surface functionalities. The signals centered at 3457, 1587, and 1087 cm^−1^ correspond to the stretching vibrations of –OH, C=C, and C–N bonds, respectively, confirming the presence of these functional groups on the catalyst surface. The C–N feature indicates that N species have been incorporated into the carbon framework. The presence of surface –OH groups also gives the catalyst a more hydrophilic character, consistent with the contact-angle results shown in Fig. S5.^26^ This favorable wettability allows the electrolyte to access the catalyst surface more readily, which may promote water adsorption and improve interfacial mass transport under HER conditions.^27^

X-ray photoelectron spectroscopy (XPS) was used to analyze the near-surface region chemical compositions and electronic states of catalysts. The XPS survey spectrum presented in Fig. S6 reveals the characteristic signals of C, O, N, Mn, and Ru, indicating the elemental composition of the catalyst surface. The Ru 3d XPS spectra (Fig. 2a) exhibit peaks at 280.5 eV and 282.4 eV, related to metallic Ru and RuO_*x*_, respectively. Notably, the Ru 3d peaks in the Ru_AC_/Mn_SA_NC catalyst undergo a negative binding energy shift relative to the Ru_NP_/NC sample. However, the Mn signals in Ru_AC_/Mn_SA_NC (Fig. 2b) exhibit a positive shift compared to those in Mn_SA_NC. This reciprocal trend indicates strong electronic coupling between Ru and Mn, likely due to partial electron transfer from Mn to Ru. Such charge redistribution modulates the electronic states of both metals, which can optimize the adsorption energies of reaction intermediates and consequently improve catalytic performance.^28^ The C 1s spectrum in Fig. 2a can be deconvoluted into four components centered at 284.8, 285.8, 286.4, and 287.4 eV, corresponding to C=C, C–N, C=O, and O–C=O species, respectively. The C–N contribution provides evidence for the incorporation of N into the carbon framework.^29^ The N 1s XPS spectrum (Fig. 2c) is fitted into two peaks, related to the pyridinic N and graphitic N. Especially, the Ru_AC_/Mn_SA_NC and Mn_SA_NC catalysts present an additional peak relative to Ru_NP_/NC, which is attributed to MnN_*x*_ moieties, confirming the formation of single atom coordination between Mn and N atoms.^30^ Furthermore, the O 1s XPS spectrum of the as-prepared catalysts can be deconvoluted into three components, corresponding to oxygen-containing functional groups, C–O bonds, and water molecules adsorbed on the surface (Fig. S7). It is evident that the introduction of Mn single atoms enhances the peak intensity associated with surface-adsorbed water in Ru_AC_/Mn_SA_NC compared to that in Ru_NP_/NC which can be ascribed to the oxophilic characteristics of Mn species.

**Fig. 2:**
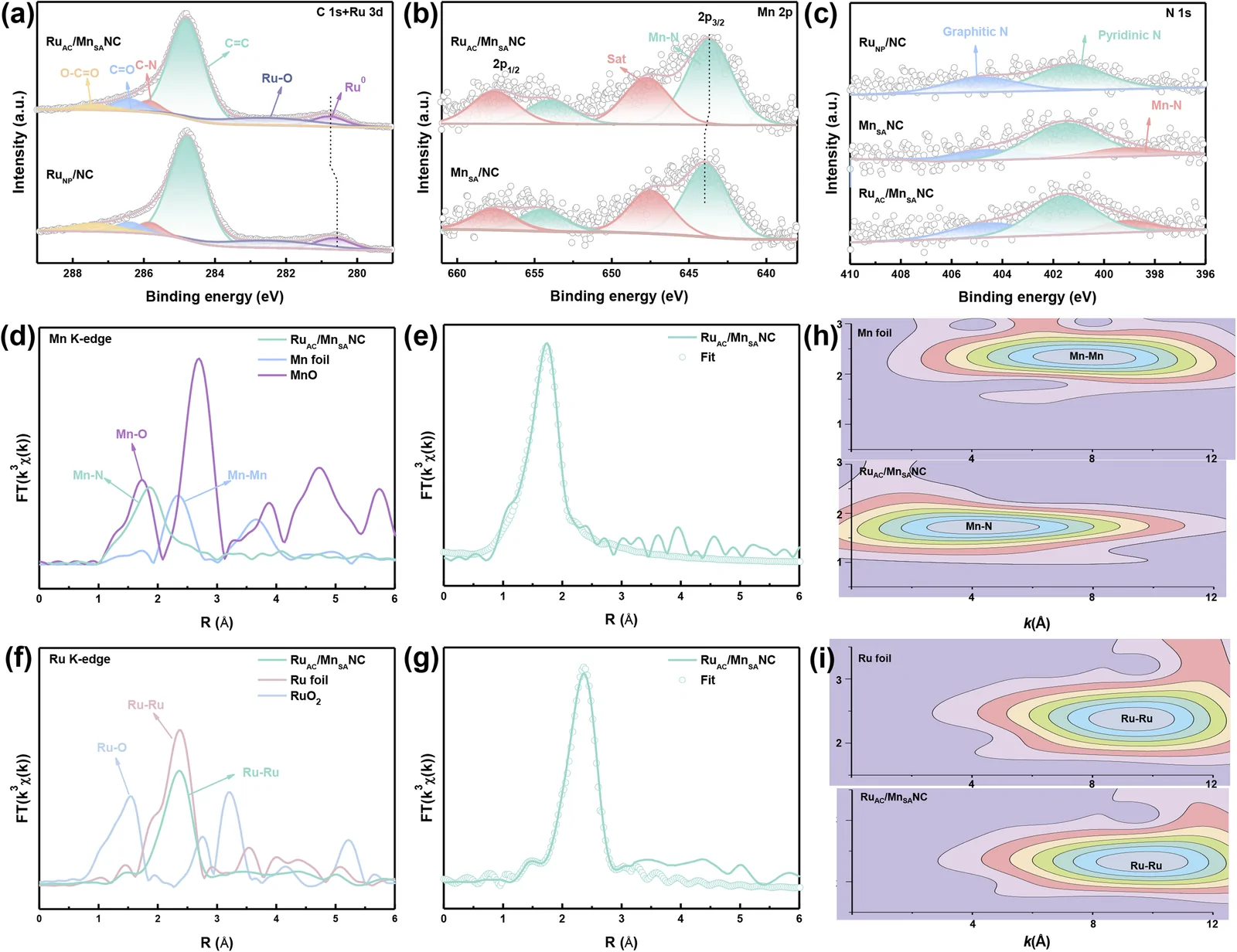
(a) Deconvoluted XPS C 1s and Ru 3d spectra of Ru_AC_/Mn_SA_NC and Ru_NP_/NC. (b) Deconvoluted XPS Mn 2p spectra of Ru_AC_/Mn_SA_NC and Mn_SA_NC. (c) Deconvoluted XPS N 1s spectra of Ru_AC_/Mn_SA_NC, Ru_NP_/NC and Mn_SA_NC. (d) Fourier-transformed *k*^3^-weighted EXAFS spectra of Ru_AC_/Mn_SA_NC on Mn K-edge. (e) Mn K-edge EXAFS spectrum of Ru_AC_/Mn_SA_NC with the corresponding fitting profile. (f) Fourier-transformed *k*^3^-weighted EXAFS spectra at the Ru K-edge. (g) Ru K-edge EXAFS spectrum of Ru_AC_/Mn_SA_NC together with its fitting profile. (h) Wavelet-transform (WT) contour plots of Ru_AC_/Mn_SA_NC and Mn foil derived from the Mn K-edge EXAFS data. (i) WT contour plots of Ru_AC_/Mn_SA_NC and Ru foil obtained from the Ru K-edge EXAFS data.

The electronic and chemical states of the Ru_AC_/Mn_SA_NC catalyst were determined by X-ray absorption near-edge spectroscopy (XANES). As revealed by the Ru K-edge XANES (Fig. S8a), the absorption profile of Ru_AC_/Mn_SA_NC is closely aligned with that of Ru foil, suggesting that Ru is primarily in a metallic state. In the Mn K-edge XANES spectra (Fig. S8b), the absorption profile of Ru_AC_/Mn_SA_NC is situated between those of Mn foil and MnO, consistent with a slightly positive charge on the Mn centers. Moreover, the local coordination environment was further elucidated by extended X-ray absorption fine structure (EXAFS) measurements. In the Fourier-transformed EXAFS profile of Ru_AC_/Mn_SA_NC, a prominent feature centered at 1.86 Å is attributable to Mn–N coordination, whereas no discernible contribution appears near 2.33 Å, suggesting the absence of Mn–Mn scattering. The absence of Mn–Mn scattering, together with the HAADF-STEM observations, supports the atomically dispersed nature of Mn species in Ru_AC_/Mn_SA_NC (Fig. 2d). Quantitative EXAFS fitting further indicates that the Mn centers are coordinated by approximately four N atoms, consistent with an Mn–N_4_ configuration embedded in the carbon matrix (Fig. 2e, S9 and Table S2).^31^ In contrast, the Fourier-transformed Ru K-edge EXAFS spectrum displays a pronounced feature at ∼2.36 Å, which is characteristic of Ru–Ru scattering in metallic Ru. This assignment is consistent with the corresponding EXAFS fitting results (Fig. 2f and g), supporting the presence of metallic Ru clusters in the Ru_AC_/Mn_SA_NC composite.^32^ Wavelet transform (WT) analysis was further performed to distinguish the local coordination environments of Mn and Ru. For the Mn K-edge, Ru_AC_/Mn_SA_NC exhibits a dominant WT intensity centered at ∼4.1 Å^−1^, distinctly different from the Mn–Mn scattering contribution observed at ∼7.7 Å^−1^ for Mn foil, in agreement with the isolated Mn configuration (Fig. 2h). At the Ru K-edge, the WT contour of Ru_AC_/Mn_SA_NC shows a distribution comparable to that of metallic Ru foil, further supporting the presence of Ru–Ru coordination within the Ru clusters (Fig. 2i). Collectively, these results establish a dual-site architecture comprising atomically dispersed Mn–N_4_ centers and metallic Ru clusters, providing the structural basis for interfacial cooperation and H relay in Ru_AC_/Mn_SA_NC.

The electrocatalytic HER performance was evaluated in 1.0 M KOH, with commercial Pt/C serving as the benchmark catalyst. As shown in Fig. 3a, Ru_AC_/Mn_SA_NC delivers substantially higher current densities than Ru_NP_/NC, Mn_SA_NC, and Pt/C over the investigated potential range. Notably, only 66 mV of overpotential is required to reach 50 mA cm^−2^ for Ru_AC_/Mn_SA_NC, compared with 133, 328, and 145 mV for Ru_NP_/NC, Mn_SA_NC, and Pt/C, respectively (Fig. 3b). These results highlight the pronounced enhancement in alkaline HER activity arising from the incorporation of atomically dispersed Mn sites and Ru clusters. The reaction kinetics were further examined using Tafel analysis. Ru_AC_/Mn_SA_NC gives a Tafel slope of 24.3 mV dec^−1^, considerably smaller than those of Ru_NP_/NC (32.5 mV dec^−1^), Mn_SA_NC (122.1 mV dec^−1^), and Pt/C (32.7 mV dec^−1^) (Fig. 3c), indicating more rapid HER kinetics on the dual-site catalyst. The low Tafel slope is consistent with a favorable Volmer–Tafel pathway, although the detailed reaction process is further discussed below based on the spectroscopic and theoretical results.^33^ Moreover, Ru_AC_/Mn_SA_NC compares favorably with recently reported Ru-based HER catalysts, exhibiting a lower *η*_50_ and a smaller Tafel slope (Fig. 3d and Table S4). These results demonstrate the competitive alkaline HER performance of the Ru_AC_/Mn_SA_NC catalyst.

**Fig. 3:**
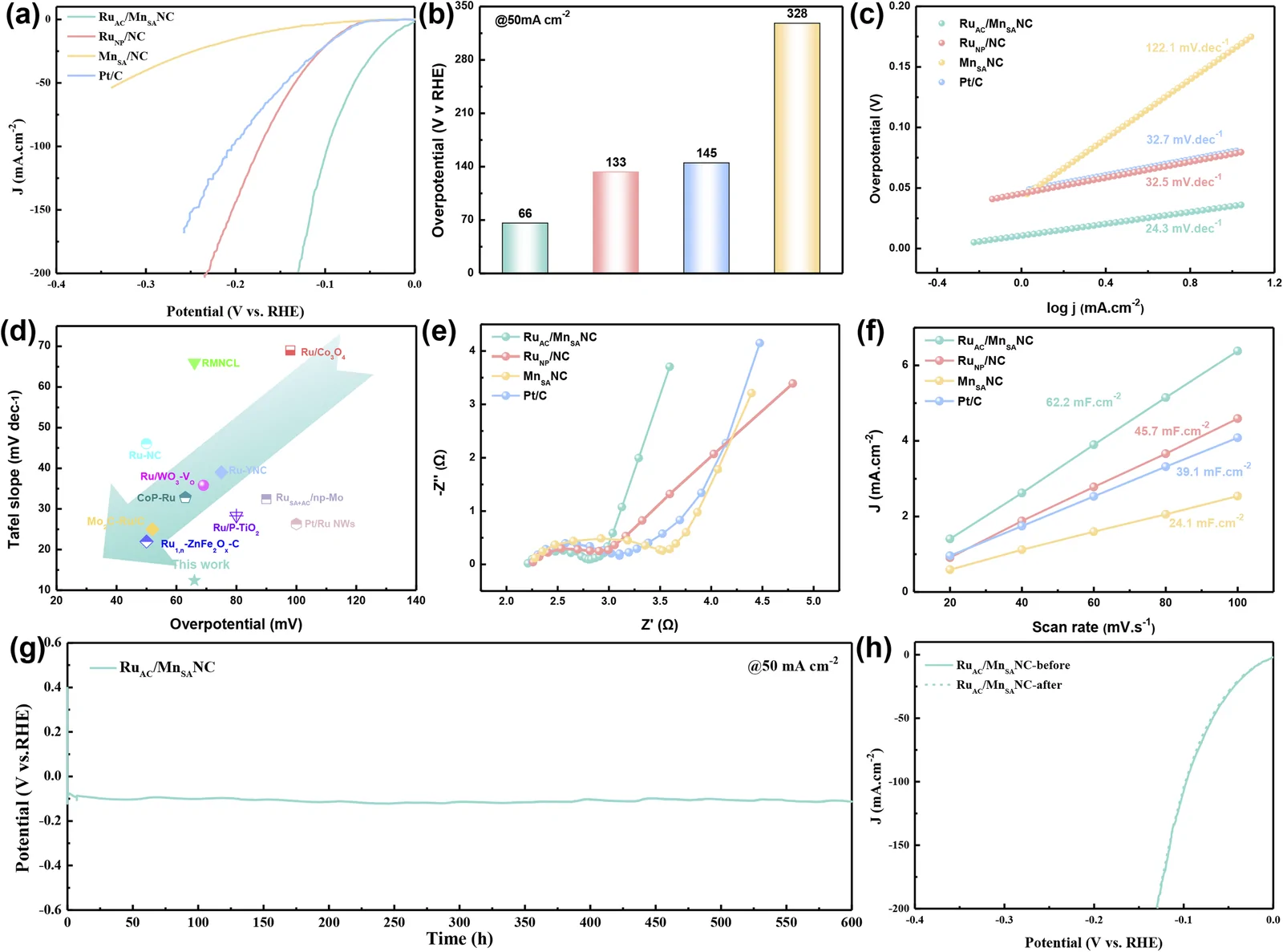
(a) LSV profiles of Ru_AC_/Mn_SA_NC, Ru_NP_/NC, Mn_SA_NC, and Pt/C. (b) Overpotentials and (c) Tafel slopes of the corresponding catalysts. (d) Comparison of the HER performance of Ru_AC_/Mn_SA_NC with recently reported catalysts. (e) Electrochemically active surface area (ECSA) derived from *C*_dl_ values and (f) Nyquist plots of the catalysts. (g) Chronopotentiometry stability profile of Ru_AC_/Mn_SA_NC. (h) LSV profiles recorded before and after the stability test.

Electrochemical impedance spectroscopy (EIS) was employed to examine the interfacial charge-transfer behavior of the catalysts. As presented in Fig. 3e, Ru_AC_/Mn_SA_NC exhibits the smallest semicircle in the Nyquist plot, corresponding to the lowest charge-transfer resistance (*R*_ct_) among the investigated samples. This result suggests more facile electron transport across the catalyst/electrolyte interface.^34^ The electrochemically active surface area (ECSA) was estimated from the double-layer capacitance (*C*_dl_) obtained from CV measurements at different scan rates (Fig. S10). Ru_AC_/Mn_SA_NC delivers a *C*_dl_ of 62.2 mF cm^−2^, markedly higher than those of Ru_NP_/NC (45.7 mF cm^−2^), Mn_SA_NC (24.1 mF cm^−2^), and Pt/C (39.1 mF cm^−2^) (Fig. 3f), indicating a larger accessible electrochemical surface area and greater exposure of catalytically accessible sites (Fig. S11). To distinguish the contribution of surface area from the intrinsic catalytic activity, the current densities were further normalized to the ECSA (*j*_ECSA_). As shown in Fig. S12, Ru_AC_/Mn_SA_NC exhibits a higher ECSA-normalized current density than the reference catalysts, suggesting that its enhanced HER activity cannot be attributed solely to the increased surface area. Instead, the enhanced intrinsic activity is therefore associated with the complementary catalytic roles of Mn single atoms and Ru clusters, which are further elucidated through *operando* Raman spectroscopy and DFT calculations below.^33^ To further evaluate the utilization efficiency of Ru, the catalytic activity was normalized to the Ru mass determined by ICP analysis and the actual catalyst loading on the working electrode (Fig. S13). Based on the ICP-determined Ru contents of 9.87 wt% for Ru_AC_/Mn_SA_NC and 10.09 wt% for Ru_NP_/NC, the Ru masses deposited on the electrodes were calculated to be 0.00823 and 0.00841 mg, respectively. At an overpotential of 100 mV, Ru_AC_/Mn_SA_NC delivered a Ru mass activity of approximately 0.94 A mg_Ru_^−1^, which was approximately 5.3 times that of Ru_NP_/NC (0.18 A mg_Ru_^−1^). The substantially enhanced Ru-mass-normalized activity demonstrates that the superior HER performance of Ru_AC_/Mn_SA_NC does not arise from a higher Ru loading but reflects the more efficient utilization of Ru enabled by the cooperative Mn–Ru interfacial structure.

Long-term operational stability is an important consideration for practical HER applications. The durability of the catalysts was first examined by chronopotentiometry at a constant current density of −50 and −100 mA cm^−2^. As shown in Fig. 3g and S14, Ru_AC_/Mn_SA_NC exhibits a nearly constant potential over 600 h at −50 mA cm^−2^ and 400 h at −100 mA cm^−2^, with no appreciable performance decay. Under identical conditions, the reference catalysts show more pronounced potential fluctuations (Fig. S15), highlighting the superior durability of Ru_AC_/Mn_SA_NC. Consistently, the LSV curve recorded after the durability test nearly overlaps with the initial polarization curve (Fig. 3h), further demonstrating the retention of its catalytic activity (Fig. S16). The excellent durability of Ru_AC_/Mn_SA_NC is also supported by its chemical and structural integrity after prolonged electrolysis. ICP analysis reveals only a minor variation in the Ru content before and after the stability test (Table S1), suggesting negligible Ru dissolution. Moreover, aberration-corrected STEM and TEM images show that the metallic species remain uniformly dispersed after electrolysis, with no obvious aggregation or structural reconstruction (Fig. S17a and b). The XRD pattern collected after the stability test also shows no discernible changes in the diffraction features (Fig. S17c). Taken together, the electrochemical, compositional, and structural characterization experiments indicate that Ru_AC_/Mn_SA_NC can effectively preserve its active structure during prolonged alkaline HER operation.

To distinguish the contributions of support activation and Mn–Ru cooperation, Ru_AC_/Mn_SA_NC-without KOH and Ru_AC_/NC were prepared as reference catalysts. In the absence of KOH activation, Ru_AC_/Mn_SA_NC-without KOH exhibited a poorly developed pore structure, a lower BET surface area, and a lower *I*_D_/*I*_G_ ratio of 1.00 than Ru_AC_/Mn_SA_NC (1.53), demonstrating that KOH activation promotes pore formation and introduces additional carbon defects (Fig. S18). Consistently, its *C*_dl_ was only 27.2 mF cm^−2^, compared with 62.2 mF cm^−2^ for Ru_AC_/Mn_SA_NC. Consequently, Ru_AC_/Mn_SA_NC-without KOH required an overpotential of 169 mV to deliver 50 mA cm^−2^, substantially higher than that of Ru_AC_/Mn_SA_NC (66 mV), confirming the beneficial role of the porous, defect-rich support in exposing accessible active sites (Fig. S19). Nevertheless, Ru_AC_/NC, despite being prepared with KOH activation and exhibiting a *C*_dl_ of 38.1 mF cm^−2^, remained markedly less active than Ru_AC_/Mn_SA_NC. Thus, the superior activity of Ru_AC_/Mn_SA_NC cannot be attributed solely to its pore structure or accessible surface area and instead reflects an additional catalytic contribution arising from the coupled Mn–N_4_ and Ru sites.

*In situ* electrochemical Raman spectra were obtained at different overpotentials in a 1.0 M KOH solution to elucidate the origin of the accelerated HER kinetics and probe the interfacial behavior of H intermediates. As revealed by the 2D contour plots of *in situ* Raman spectra (Fig. 4a and b), the Ru_AC_/Mn_SA_NC catalyst shows a significantly increased interfacial water signal and a faster depletion rate compared to the Ru_NP_/NC catalyst. This enhancement is ascribed to the synergistic effects between Ru clusters and oxophilic Mn single atoms, which together facilitate sufficient interfacial water availability and enhance the kinetics of water dissociation during the alkaline hydrogen evolution reaction (HER) (Fig. 4c).^20^ In addition, the Ru_AC_/Mn_SA_NC catalyst exhibits a notably more intense Raman band around 1921 cm^−1^ compared to its Ru_NP_/NC counterpart, which can be assigned to the Ru(*n*+)–H* stretching vibration associated with hydrogen adsorbed on Ru sites.^35^ The enhanced intensity of this band in Ru_AC_/Mn_SA_NC signifies a higher surface coverage of the key H* intermediate during the HER, which is ascribed to a synergistic mechanism facilitated by the introduction of Mn single atoms. Mn, known for its oxophilic nature, acts as a preferential site for water adsorption and activation. The inherent oxophilicity of Mn enables more efficient water dissociation than the Ru_NP_/NC surface. The enhanced H coverage on Ru, together with the preferential water activation at Mn sites, supports an interfacial H relay from Mn to adjacent Ru clusters. This continuous relay of H from Mn to Ru not only enriches H coverage on Ru but also regenerates the Mn sites for subsequent water dissociation. Consequently, this synergistic cycle significantly accelerates the overall HER kinetics. To further verify the above-proposed interfacial H relay scenario, we performed isotope-labeling DEMS characterization experiments combined with *operando* Raman measurements. As shown in Fig. S20, Ru_AC_/Mn_SA_NC exhibits pronounced, reproducible responses at *m*/*z* = 2 (H_2_) and *m*/*z* = 3 (HD) during repeated electrochemical cycles, whereas the corresponding signals from Ru_NP_/NC are substantially weaker. The HD response of Ru_AC_/Mn_SA_NC progressively decreases over successive cycles, consistent with the gradual consumption or exchange of retained deuterium during hydrogen evolution in H_2_O. The enhanced HD response upon Mn incorporation supports the involvement of an exchangeable hydrogen pool associated with the coupled Mn–Ru architecture. Combined with the *operando* Raman observation of Ru-bound H*, this isotope-labeling result provides additional experimental support for the proposed participation of Mn-associated sites in supplying hydrogen to neighboring Ru sites.

**Fig. 4:**
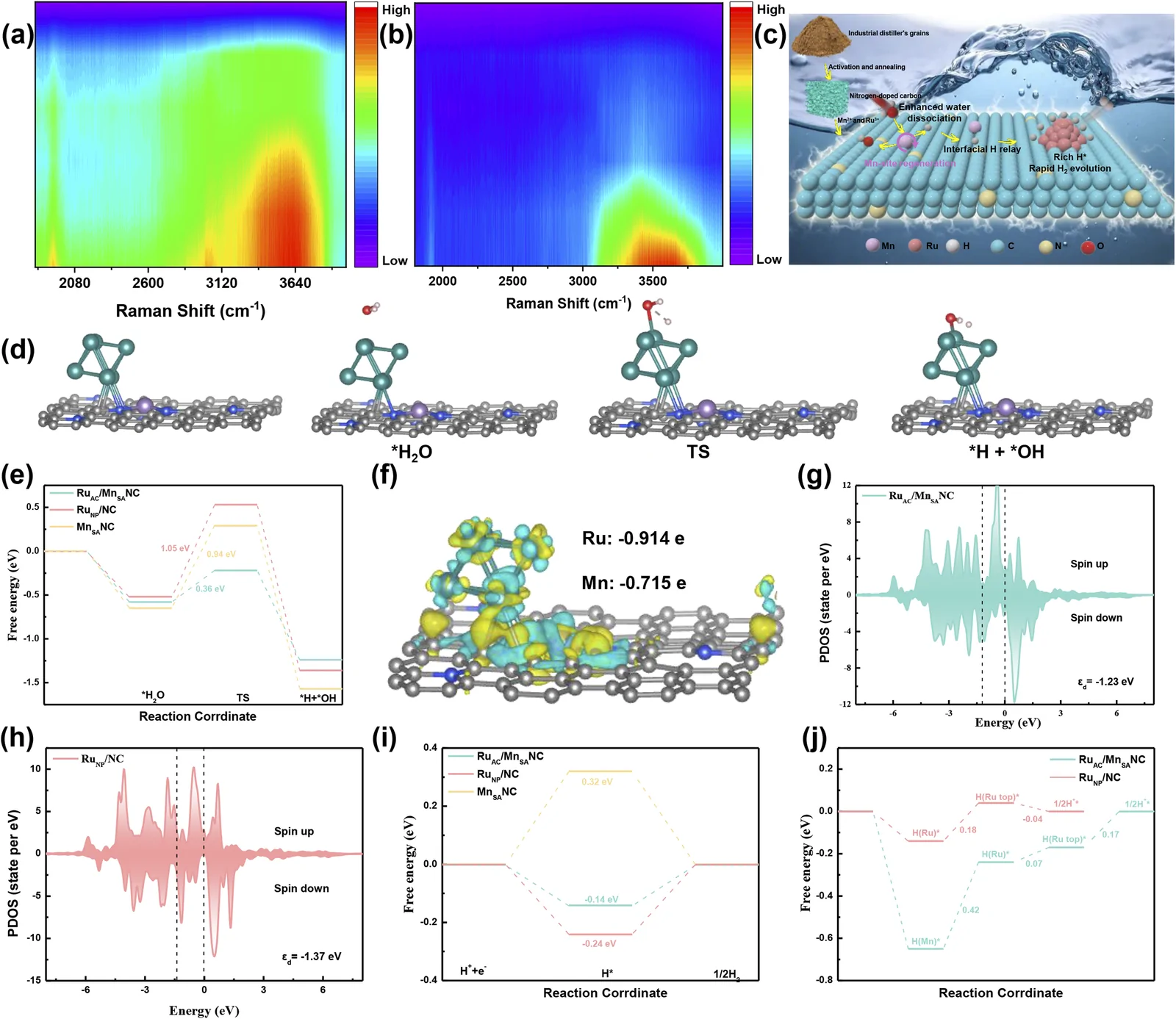
2D contour plots from *operando* Raman spectroscopy of (a) Ru_AC_/Mn_SA_NC and (b) Ru_NP_/NC. (c) Schematic of the alkaline HER process over the Ru_AC_/Mn_SA_NC catalyst. (d) Process schematic of the HER on Ru_AC_/Mn_SA_NC under alkaline conditions. (e) Gibbs free energy profiles for the H-down water dissociation pathways on Ru_AC_/Mn_SA_NC, Ru_NP_/NC, and Mn_SA_NC surfaces, depicting the reactant, transition (TS), and product states. (f) Charge density images of Ru_AC_/Mn_SA_NC. (g and h) Calculated PDOS of Ru sites on Ru_AC_/Mn_SA_NC and Ru_NP_/NC. (i) Calculated Gibbs free energy profile on the Ru site in Ru_AC_/Mn_SA_NC, Ru_NP_/NC, and Mn_SA_NC. (j) HER through interfacial H relay from a Mn single atom to a Ru cluster for the Ru_AC_/Mn_SA_NC system.

Density functional theory (DFT) computations were carried out to investigate the intrinsic mechanisms that improve the HER performance of the Ru_AC_/Mn_SA_NC catalyst in alkaline media. Based on experimental characterization, optimized models of Ru_AC_/Mn_SA_NC, Ru_NP_/NC, and Mn_SA_NC incorporating carbon defects in the N-doped carbon support were constructed for comparison (Fig. S21). In Ru_AC_/Mn_SA_NC, a Mn–N_4_ site is coupled to a finite Ru cluster, with their placement guided by the mean projected separation of 3.5 Å from four aberration-corrected electron microscopy measurements (Fig. S22). This model represents an experimentally informed local configuration rather than the full structural heterogeneity of the catalyst. As shown in Fig. 4d, e and S23, the calculated water-dissociation barriers on Ru_NP_/NC and Mn_SA_NC are 1.05 and 0.94 eV, respectively. The lower barrier on Mn_SA_NC indicates that the isolated oxophilic Mn–N_4_ site facilitates water dissociation more effectively than the Ru_NP_/NC surface. Notably, the barrier is further reduced to only 0.36 eV at the coupled Mn–Ru interface of Ru_AC_/Mn_SA_NC. These results demonstrate that Mn promotes water activation and that its interfacial coupling with the adjacent Ru cluster provides a more favorable configuration for H_2_O dissociation into adsorbed H* and OH* intermediates.^36^ The calculated charge density distributions are applied to reveal the interfacial electronic structure between Ru and the Mn_SA_NC substrate in the Ru_AC_/Mn_SA_NC system. As revealed in Fig. S24, the Mn atom in Mn_SA_NC carries a substantial negative charge (−0.951*e*), reflecting its electron-rich character on the N-doped carbon support. The Ru nanoparticle in Ru_NP_/NC also shows significant electron accumulation (−0.827*e*). Notably, the charge redistribution of the Ru_AC_/Mn_SA_NC catalyst occurs distinctly: the electron density on the Ru cluster increases to −0.914*e*, while Mn remains highly charged (−0.715*e*) (Fig. 4f). This trend confirms electron transfer from Mn to the electron-accepting Ru site, resulting in an increase in the electron density of Ru active centers.^37^ This result usually causes the d-band center to shift upward, thereby weakening its adsorption strength for the reaction intermediate (H*), which is beneficial for the subsequent hydrogen desorption step and ultimately enhances the activity of the HER.^38^ To gain a deeper insight into the electronic structures of Ru and Mn arising from the electron transfer and charge redistribution, the projected density of states (PDOS) was computed. As shown in Fig. 4g and h, the computed d-band center of Ru clusters in Ru_AC_/Mn_SA_NC (1.23 eV) shows a notable decrease relative to that of Ru_NP_/NC (1.37 eV), supporting the notion that the inclusion of Mn single atoms reduces the excessive Ru–H bond strength, thereby facilitating more efficient H* transfer and H_2_ desorption.^39^ The computed Δ*G*_H*_ value for Ru_NP_/NC is −0.24 eV, indicating a high degree of H* adsorption on the metallic Ru site surfaces. In contrast, the integration of Mn single atoms significantly optimizes the Δ*G*_H*_ on Ru sites within Ru_AC_/Mn_SA_NC, reducing it to −0.14 eV and consequently promoting rapid kinetics for alkaline HER (Fig. 4i). The calculated free-energy profile further reveals a favorable H relay from the Mn site to the adjacent Ru cluster (Fig. 4j). H is preferentially stabilized at the Mn site following water dissociation and subsequently transfers to the Ru cluster, where the near-optimal hydrogen adsorption thermodynamics favor H_2_ formation and desorption. This energetically favorable interfacial relay provides a molecular-level explanation for the complementary roles of Mn and Ru sites during alkaline HER.

## 4. Conclusion

In summary, we have successfully constructed a high-performance and durable Ru_AC_/Mn_SA_NC electrocatalyst by co-anchoring Ru clusters and Mn single atoms on a N-doped carbon substrate for the alkaline hydrogen evolution reaction (HER). Comprehensive experimental and theoretical analyses reveal an interfacial H relay between Mn single atoms and Ru clusters, where oxophilic Mn sites promote water dissociation and the generated H intermediates are subsequently transferred to adjacent Ru clusters with near-optimal hydrogen adsorption thermodynamics. This relay process simultaneously enriches H* on Ru sites and regenerates Mn sites for subsequent water activation, establishing an efficient catalytic cycle for alkaline HER. The optimized Ru_AC_/Mn_SA_NC catalyst delivers exceptional catalytic activity, achieving a low overpotential of merely 66 mV at a current density of 50 mA cm^−2^ in alkaline medium. More importantly, it demonstrates outstanding operational stability, retaining its performance for over 600 hours. This work not only provides a superior catalyst candidate for electrochemical hydrogen production but also offers profound atomic-level insight into the design of synergistic catalytic systems *via* a rational integration of single atoms and clusters.

## Author contributions

Shangyan Zhou: conceptualization, methodology, writing – original draft. Fei Liu: funding acquisition, supervision, writing – review & editing. Qiandan Ren: data curation. Huarui Wang: software. Yanyu Zhang: data curation. Quande Man: data curation. Xiaodan Wang: funding acquisition, supervision. Ju Guo: methodology, investigation.

## Conflicts of interest

The authors declare no competing financial interest.

## Supplementary Material

SC-OLF-D6SC06709C-s001

## Data Availability

All data supporting the findings of this study are available in the main text and the supplementary information (SI). Raw experimental data, characterization data, and computational data are available from the corresponding author upon reasonable request. Supplementary information is available. See DOI: https://doi.org/10.1039/d6sc06709c.
