# Supplementary material for: Interfacial H relay and site regeneration in Mn single atom-Ru cluster hybrids for efficient alkaline HER
Source: Chem Sci. 2026 Nov 4. Online ahead of print. doi: 10.1039/d6sc06709c (PMC13732579; doi:10.1039/d6sc06709c)
Supplement: Supplementary file 1 [file SC-OLF-D6SC06709C-s001.pdf]

Supplementary Information (SI) for Chemical Science.
This journal is © The Royal Society of Chemistry

Interfacial H Relay and Site Regeneration in Mn Single Atom–Ru Cluster Hybrids for Efficient Alkaline HER

Shangyan Zhou[a,b,c,d], Fei Liu[a,b,c,d], Qiandan Ren[a,b,c,d], Huarui Wang[a,b,c,d], Yanyu Zhang[a,b,c,d], Quande Man[a,b,c,d], Ju Guo[e]*

[a]*Department of Chemical Engineering, School of Chemistry and Chemical Engineering, Guizhou University, Guiyang, Guizhou 550025, China*

[b]*Key Laboratory of Carbon-based Energy Molecular Chemical Utilization Technology in Guizhou Province, Guizhou University, Guiyang, Guizhou 550025, China*

[c]*Guizhou Provincial Key Laboratory of Green Catalysis and Materials for Resource Conversion, Guizhou University, Guiyang, Guizhou, 550025, China*

[d]*Guizhou Provincial Technological Innovation Centre of Resource Coupling and Deep Processing for Sulfur-Phosphorus-Titanate-Iron, Guizhou University, Guiyang, Guizhou, 550025, China*

[e]*School of Brewing Engineering, Moutai Institute, Renhuai, Guizhou, 564507, China*

Corresponding Author: Fei Liu (Email: ce.feiliu@gzu.edu.cn)

Electrochemical measurements

All electrochemical tests were performed in a standard three-electrode system on a CS310X electrochemical workstation (Kesite, Wuhan) in a 1 M KOH aqueous solution at room temperature. The glassy carbon electrode (0.07 cm^2), a graphite rod and a Hg/HgO

electrode were used as the working, counter, and reference electrodes, respectively. All potentials, unless mentioned otherwise, were transformed to the reversible hydrogen electrode (RHE) according to the Nernst equation: $E_{RHE} = E_{Hg/HgO} + 0.098 + 0.059 \times pH$. The catalyst ink and working electrode were prepared as follows. 5 mg of catalyst was dispersed in 600 μL of ethanol and ultrasonicated for 30 min to form a homogeneous catalyst ink with a concentration of 8.33 mg mL^{-1}. Subsequently, 10 μL of the ink was drop-cast onto a working electrode with a geometric area of 0.07 cm2 and dried before electrochemical testing. The catalyst mass deposited on each electrode was therefore approximately 0.0833 mg, corresponding to a geometric catalyst loading of approximately 1.19 mg cm^{-2}. Linear scanning voltammetry (LSV) was performed at the potential range from -0.8 to -1.5 V vs (All data has not undergone IR compensation). Hg/HgO with a scan rate of 5 mV s^{-1}. LSV curves were manually corrected. The double-layer capacitance (C_{dl}) was calculated by performing cyclic voltammetry (CV) at non-Faradic region with different scan rates of 20, 40, 60, 80, and 100 mV s^{-1}. Electrochemical impedance spectroscopy (EIS) was tested in the frequency range from 10^5 Hz to 1 Hz with an amplitude of 5 mV. The stability of catalysts was assessed via chronopotentiometry under constant-current conditions (-50/-100 mA cm^{-2}) in the KOH aqueous solution.

The ECSAs were calculated using the following equation:

$$ECSA = C_{dl}/C_s \times S$$

where C_s corresponds to the specific capacitance and=40 μF cm^{-2} and S is the surface area of the working electrode.

The Ru mass activity was calculated using:

$$MA_{Ru} = \frac{|j| \times A}{m_{Ru}}$$

where $|j|$ is the absolute cathodic current density at a given overpotential, A is the geometric electrode area, and m_{Ru} is the ICP-determined Ru mass on the electrode.

Differential electrochemical mass spectrometry (DEMS) measurements

Isotope-labeling DEMS experiments were carried out on a home-modified differential electrochemical mass spectrometry system to trace the interfacial hydrogen transfer process. A custom-made thin-layer electrolytic cell coupled with a porous Teflon membrane interface was employed to realize real-time sampling of gaseous products under electrochemical polarization. All electrochemical measurements were performed under constant-potential mode controlled by an electrochemical workstation. For the deuterium pre-labeling procedure, the catalyst ink was drop-cast onto a glassy carbon working-electrode surface. The electrode was first subjected to continuous potentiostatic polarization in D_2O-based 1.0 M KOH electrolyte at a fixed potential for sufficient time to complete deuterium isotope exchange and pre-load deuterium-containing hydrogen intermediates on catalyst surface. After pre-labeling, the D_2O-containing electrolyte was completely removed, and the cell was carefully rinsed several times with D_2O-free ultrapure water. Subsequently, fresh H_2O-based 1.0 M KOH aqueous electrolyte was injected into the DEMS cell. Thereafter, the electrode was operated under identical constant-potential conditions for repeated cyclic testing. Mass-to-charge signals of $m/z = 2$ (H_2) and $m/z = 3$ (HD) were continuously recorded by the mass spectrometer. The time-resolved mass spectral intensity signals were synchronously collected together with electrochemical data. During successive potentiostatic cycles, the evolution and decay of HD signal originating from the exchange

between pre-adsorbed deuterium species and aqueous-phase water were monitored, which reflects the exchangeable hydrogen reservoir on the catalyst surface. All DEMS tests were performed under Ar gas atmosphere protection.

Characterizations

X-ray diffraction (XRD, D8 advance from Bruker Inc, Germany) was conducted on with Cu-Kα radiation (λ=1.5418 Å). The morphology and elements distribution of catalysts were characterized by scanning electron microscopy (SEM, VEGA-3 from TESCAN Inc, China) and transmission electron microscope (TEM, JEM-2100F) equipped with energy dispersive spectroscopy (EDS). Aberration-corrected high-angle annular dark-field scanning transmission electron microscopy (AC-HAADF-STEM) was recorded on a Thermo Fisher-Spectra 300 TEM. XPS spectra were recorded in K-Alpha Plus (Somerfield, USA) with Al-Ka X-ray as the light source and were calibrated by C 1s binding energy (284.8 eV) on a Avantage instrument. To explore the hydrophilicities of the catalysts, the water contact angles were tested with a contact angle analyzer (OCA20, Dataphysics, Germany). The N_2 adsorption/desorption and pore size analyzers are ensured using the ASAP 2020 instrument. Raman spectra were recorded using a Raman spectrometer (LabRam HR Evolution) with 532 nm visible wavelength. Fourier Transform infrared spectroscopy (FTIR) was manipulated to inspect the signals of the intermediates using a Nicolet IS50 Spectroscopy equipped with a liquid nitrogen-cooled mercury-cadmium-telluride (MCT) detector. Inductively coupled plasma mass spectrometry (ICP-MS) analysis was manipulated on an Thermo ICAP PRO instrument. X-ray absorption spectroscopy (XAS) measurements at the Ru k-edges were performed at SPring-8 (Japan Synchrotron Radiation Research Institute,

Hyogo, Japan). The samples were operated at 8.0 GeV with a constant current of 99.5 mA. The data were treated using the Athena package for energy calibration and normalization. For Wavelet Transform (WT) analysis, the χ(k) exported from Athena was imported into the Hama Fortran code.

Theoretical calculations

All spin-polarized Density Functional Theory (DFT) calculations were conducted within the Vienna Ab into Simulation Package (Vasp) [1]. Electron-ion interactions were described by projector-augmented wave (PAW) potentials [2]. Exchange-correlation effects were described by Perdew-Burke-Ernzerhof (PBE) functional of generalized gradient approximations (GGA) [3, 4]. The plane wave cutoffenergy was specified by 450 eV and electron smearing via a 2nd-order Methfessel-Paxton [5] technique with a width of 0.1 eV. The convergence criteria for electronic self-consistent interactions and forces were set to 10-5 eV and 0.05 eV/Å, respectively. The Brillouin zone sampling was performed using the Monkhorst-Packscheme [6].

The free energy was calculated using the equation:

$$G=E_{ads}+ZPE-TS$$

where G, E_{ads}, ZPE and TS are the free energy, total energy from DFT calculations, zero point energy and entropic contributions, respectively.

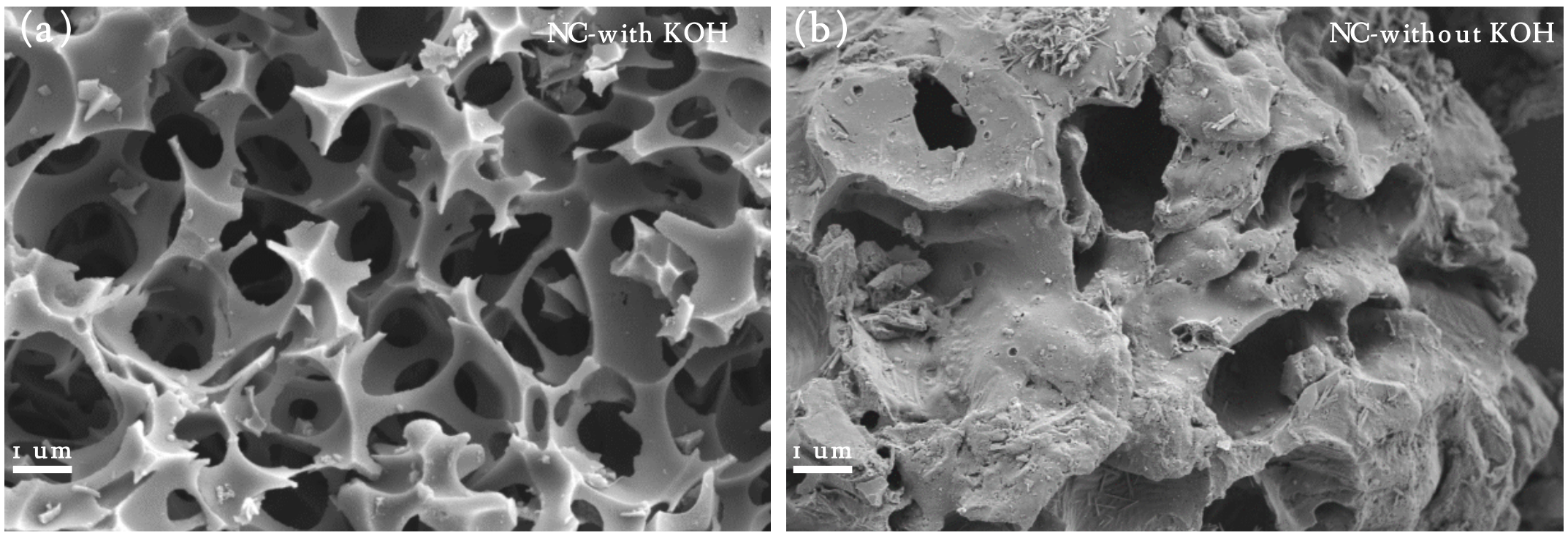

Figure S1. SEM image of NC-with KOH and NC-without KOH.

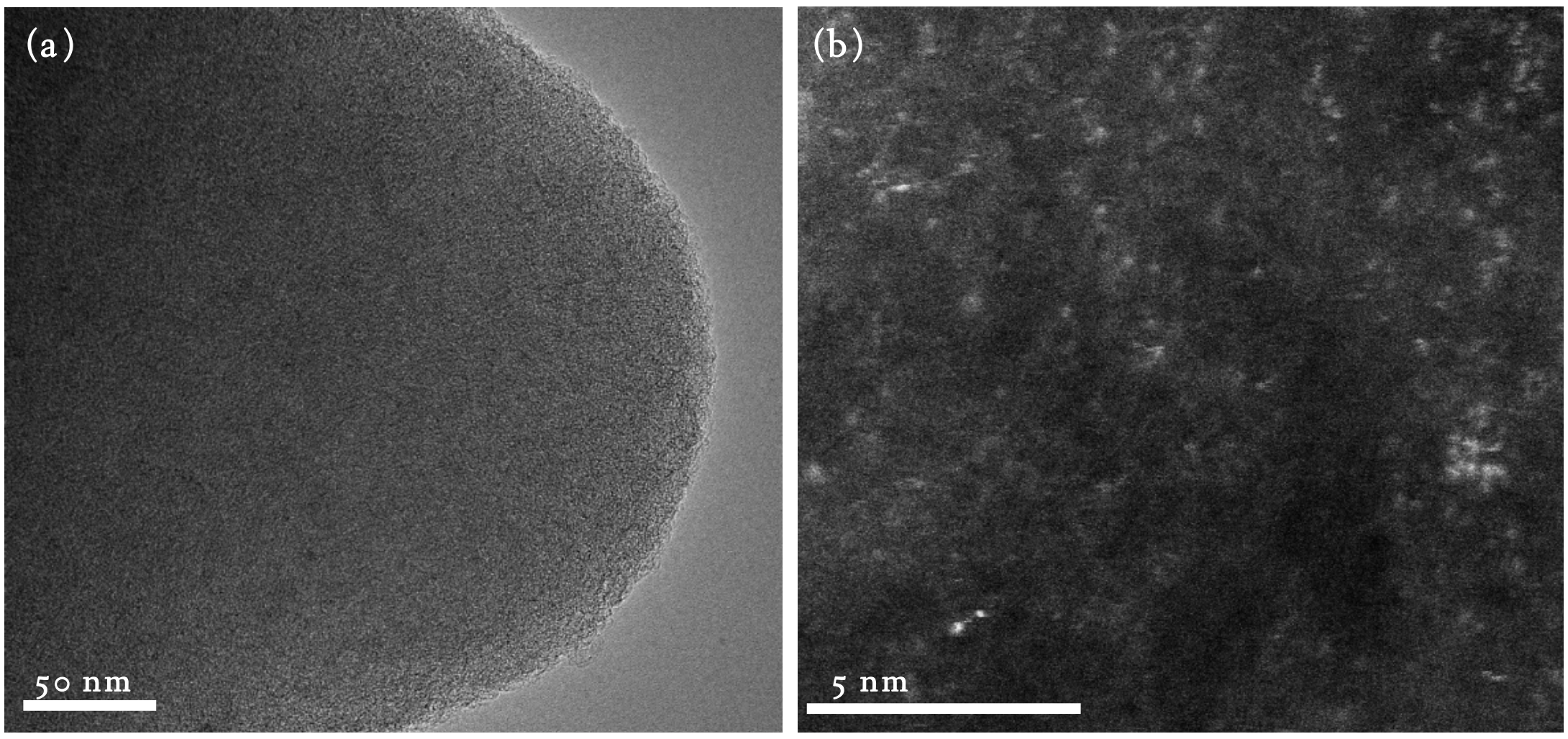

Figure S2. a) TEM image and b) aberration-corrected HAADF STEM image of $Mn_{SA}NC$.

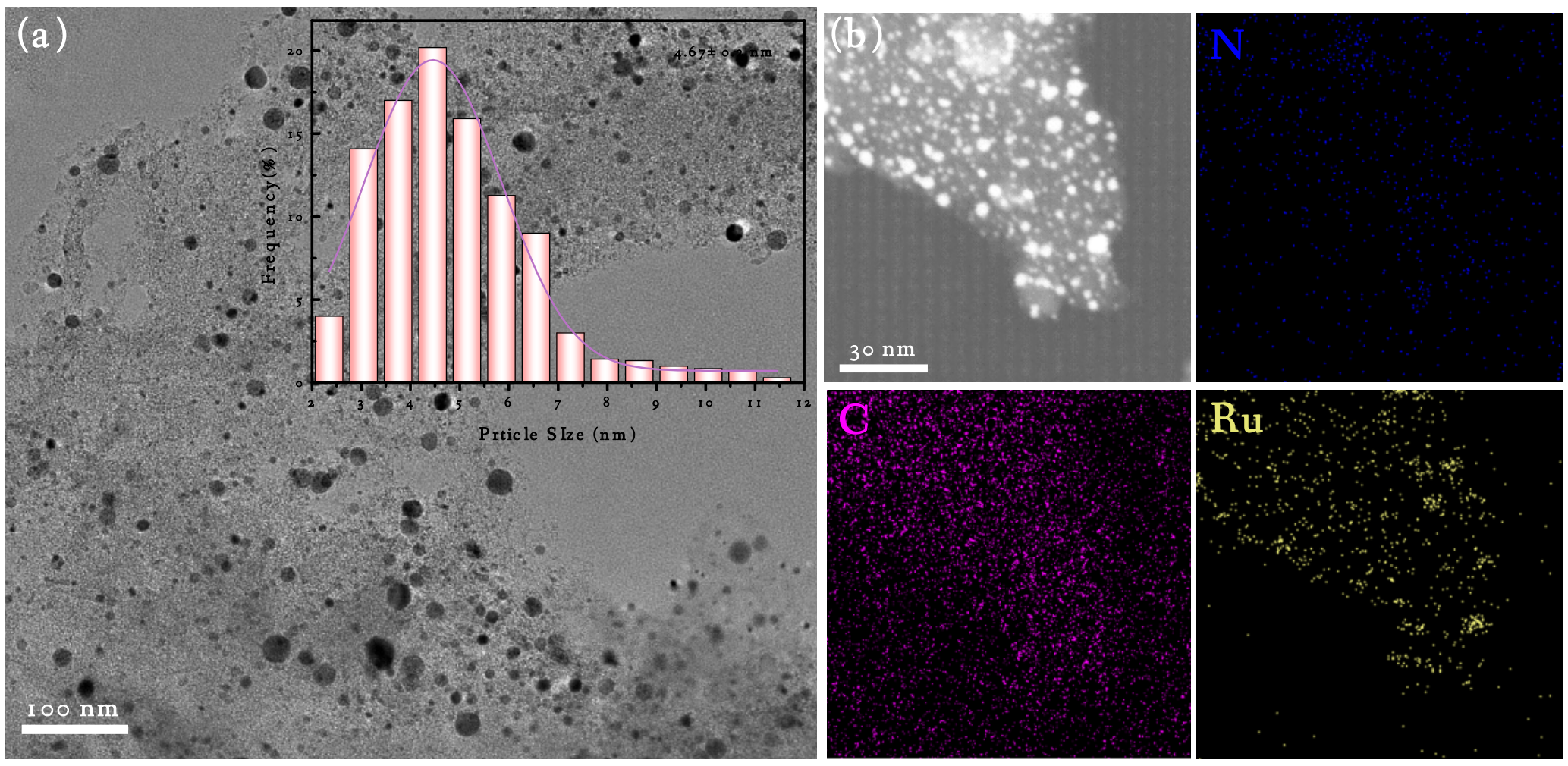

Figure S3. a) TEM image and b) HAADF-STEM and corresponding EDS spectroscopy elemental mapping of Pt_{NP}/NC.

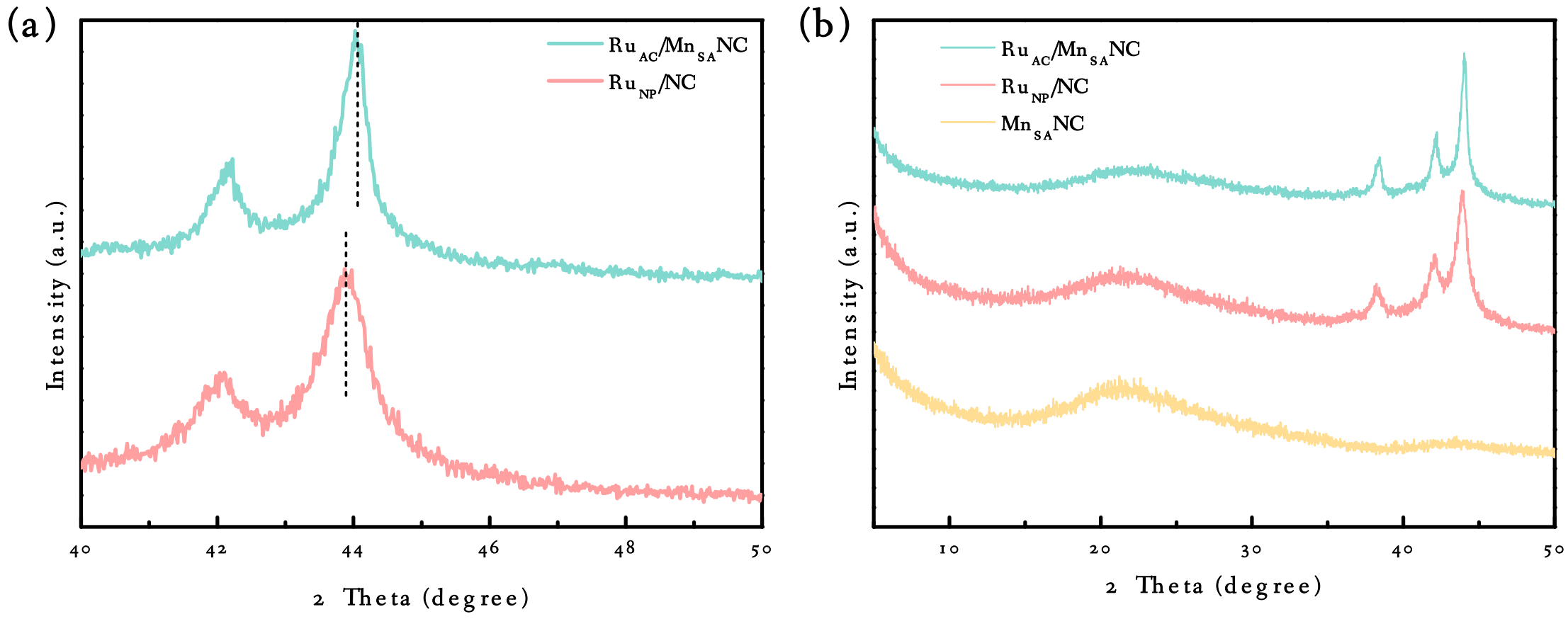

Figure S4. Localized magnified image of XRD of $Ru_{AC}/Mn_{SA}NC$.

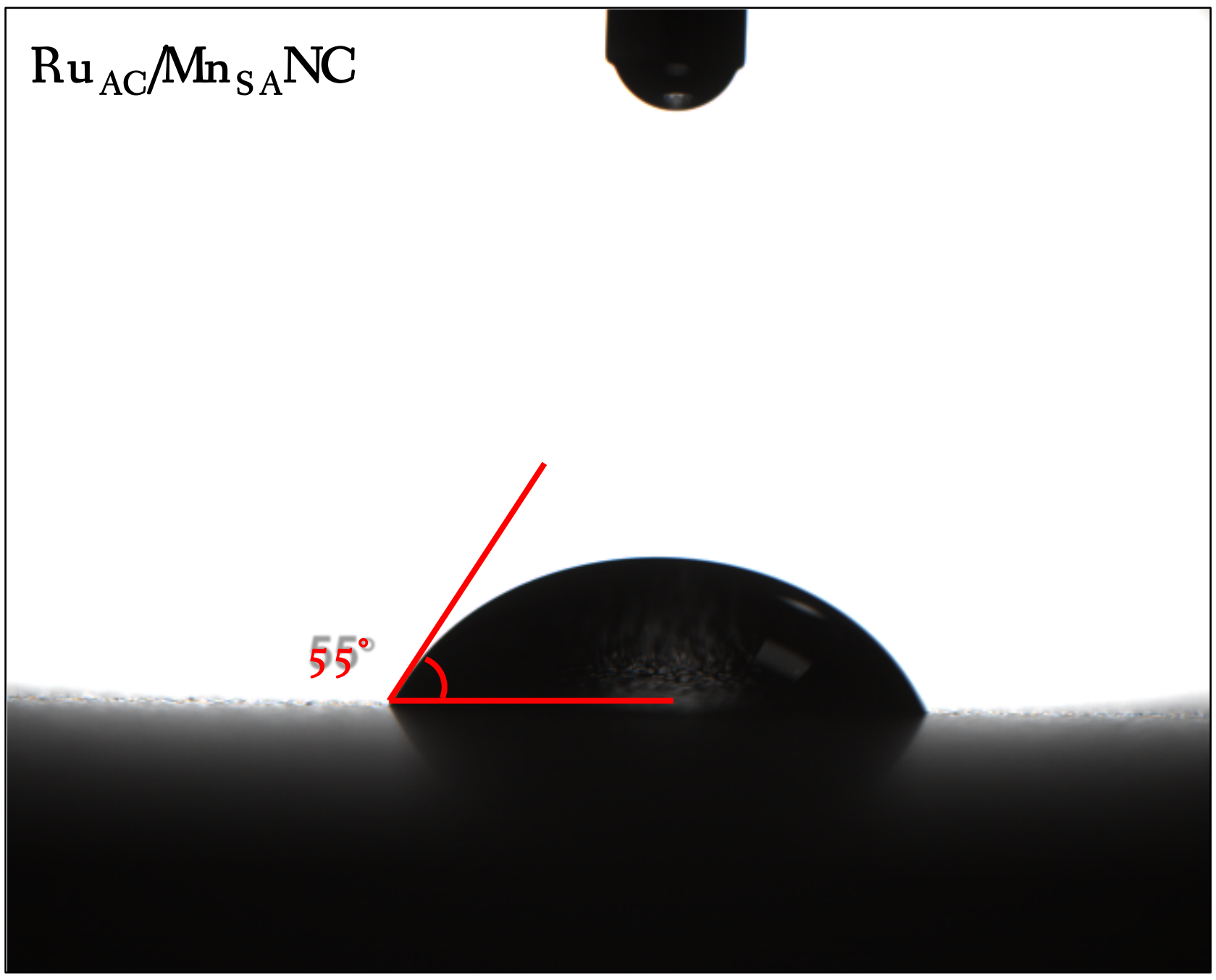

Figure S5. Contact angles of $Ru_{AC}/Mn_{SA}NC$.

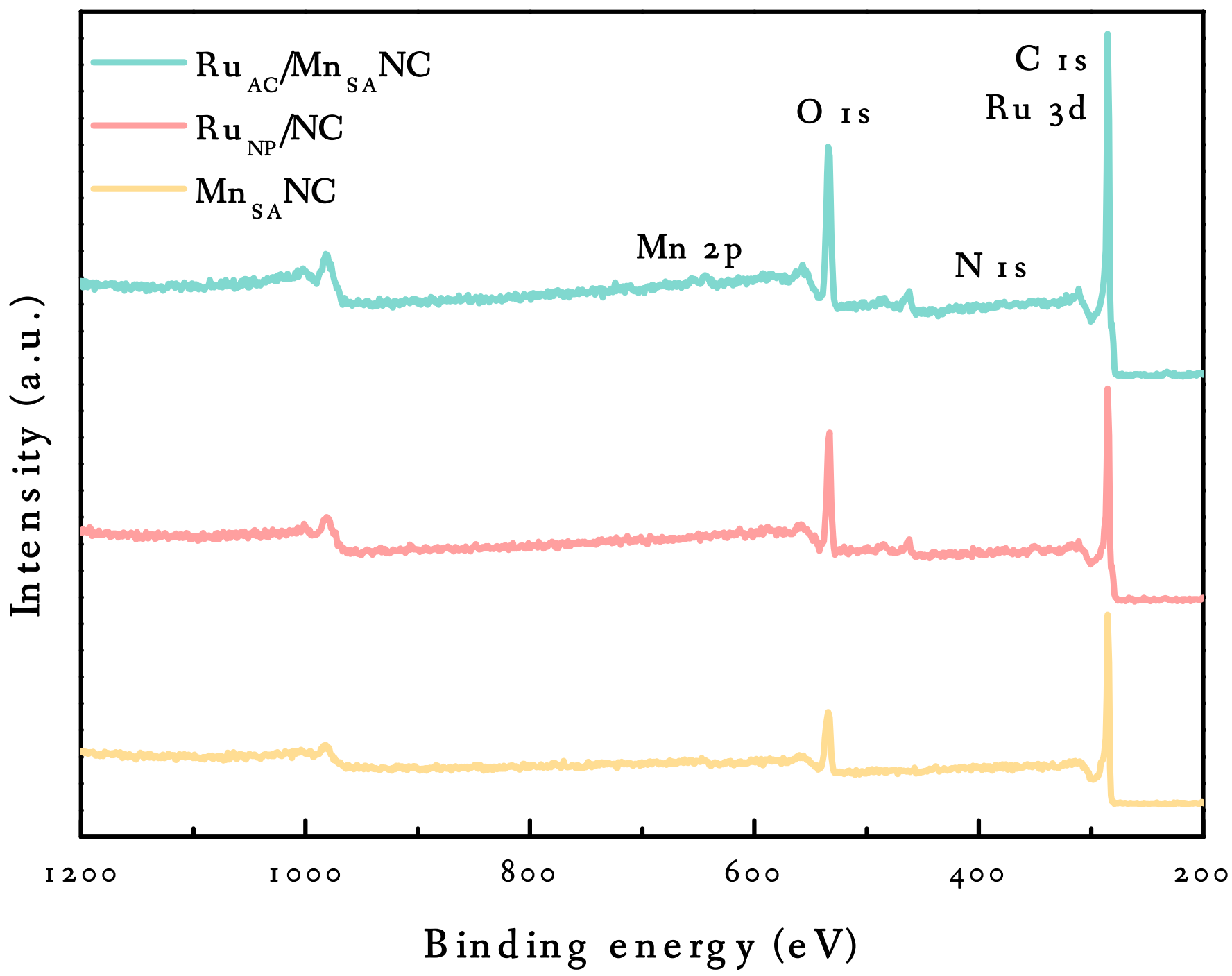

Figure S6. XPS spectra of $Ru_{AC}/Mn_{SA}NC$, Ru_{NP}/NC and $Mn_{SA}NC$.

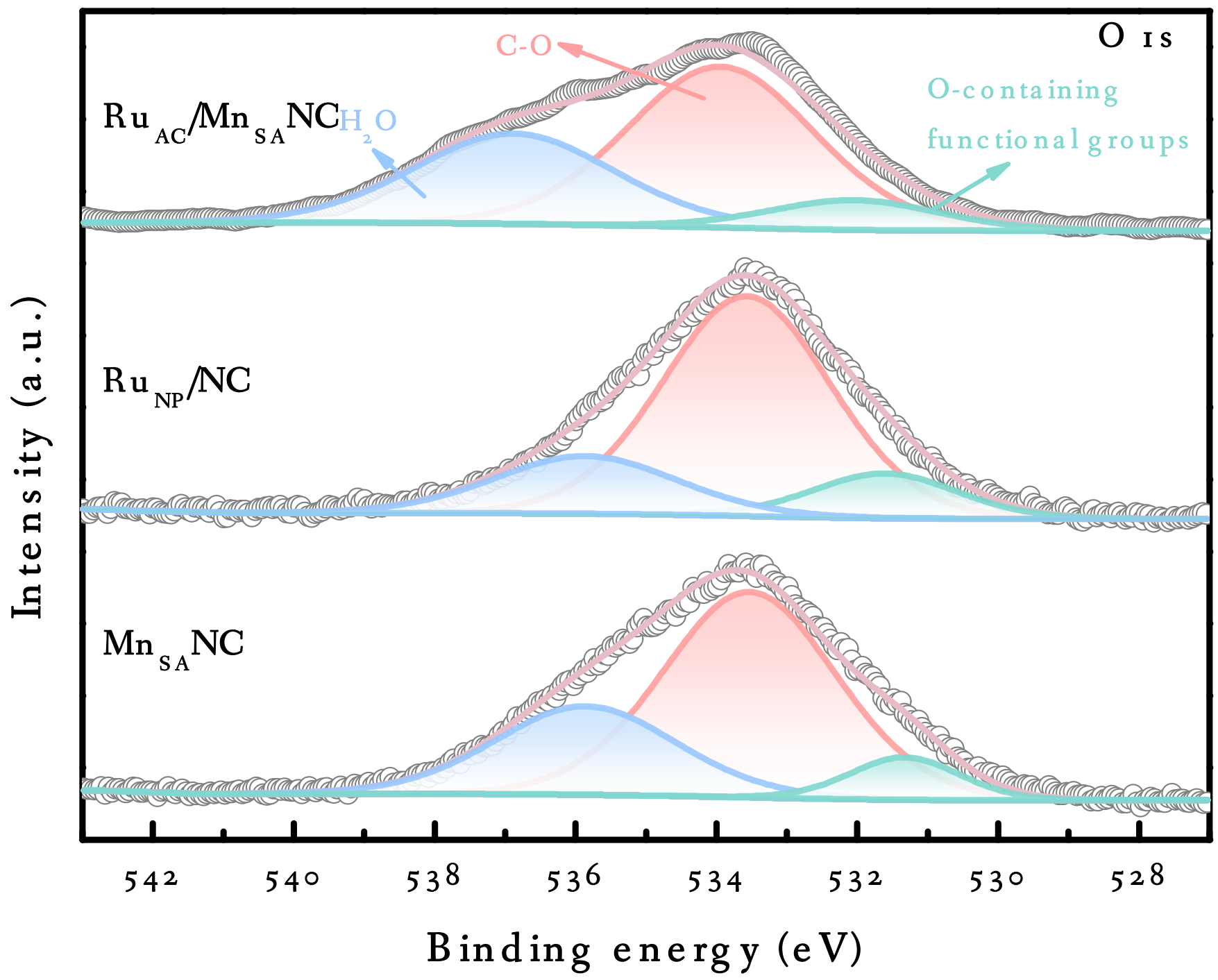

Figure S7. Deconvoluted XPS O 1s of $Ru_{AC}/Mn_{SA}NC$, Ru_{NP}/NC and $Mn_{SA}NC$.

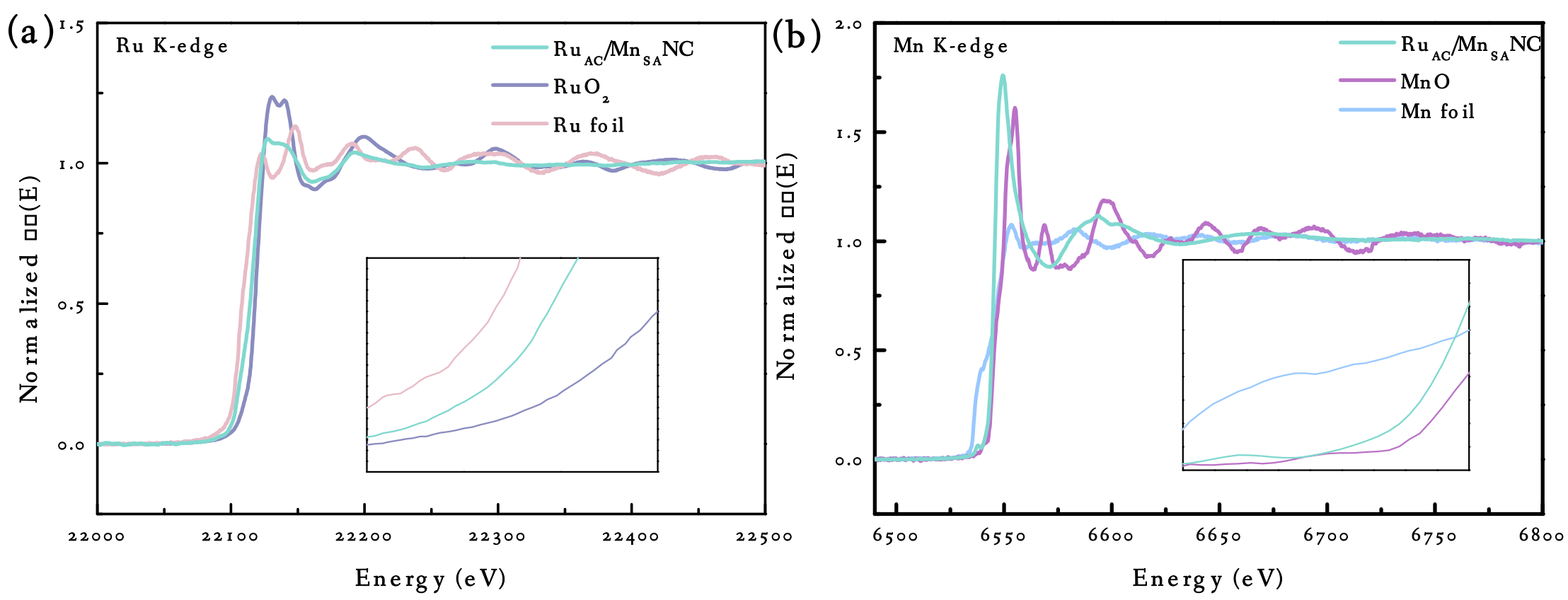

Figure S8. XANES spectra of a) Ru K-edge and b) Mn K-edge.

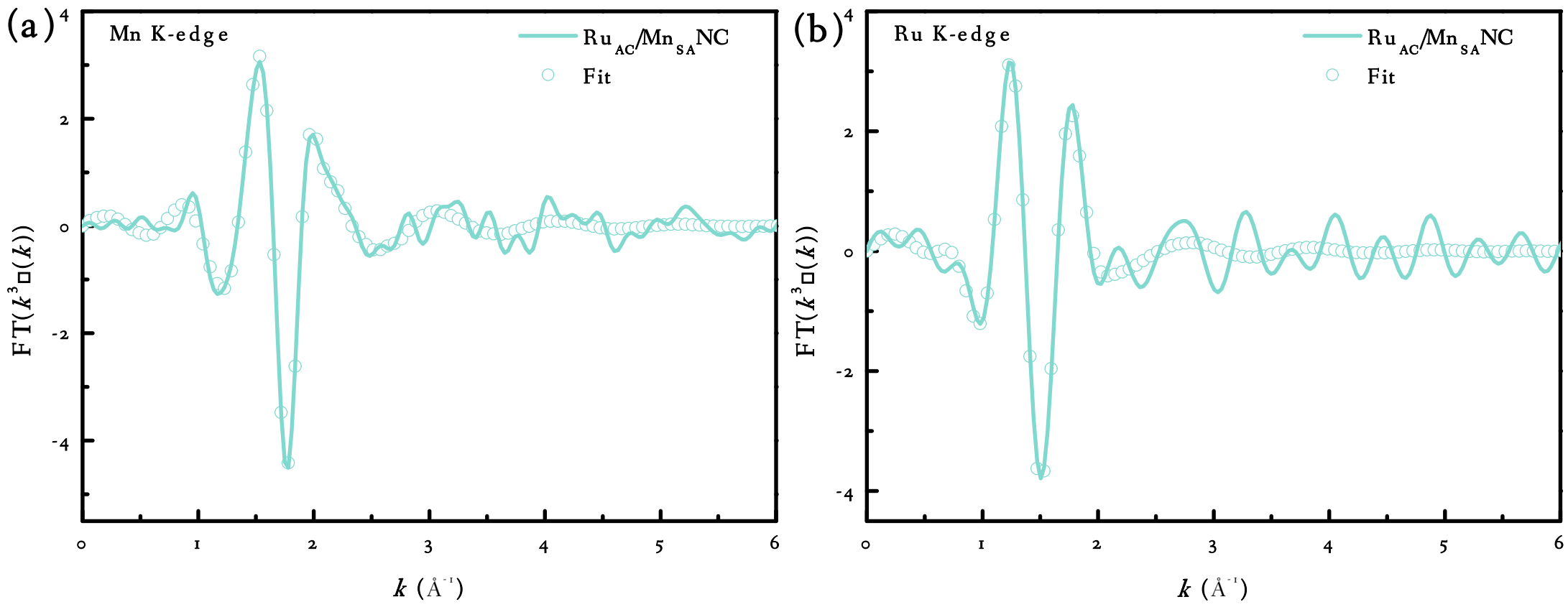

Figure S9. Experimental and fitted a) Mn K-edge EXAFS spectra and b) Ru K-edge EXAFS spectra at k^3 space of $Ru_{AC}/Mn_{SA}NC$.

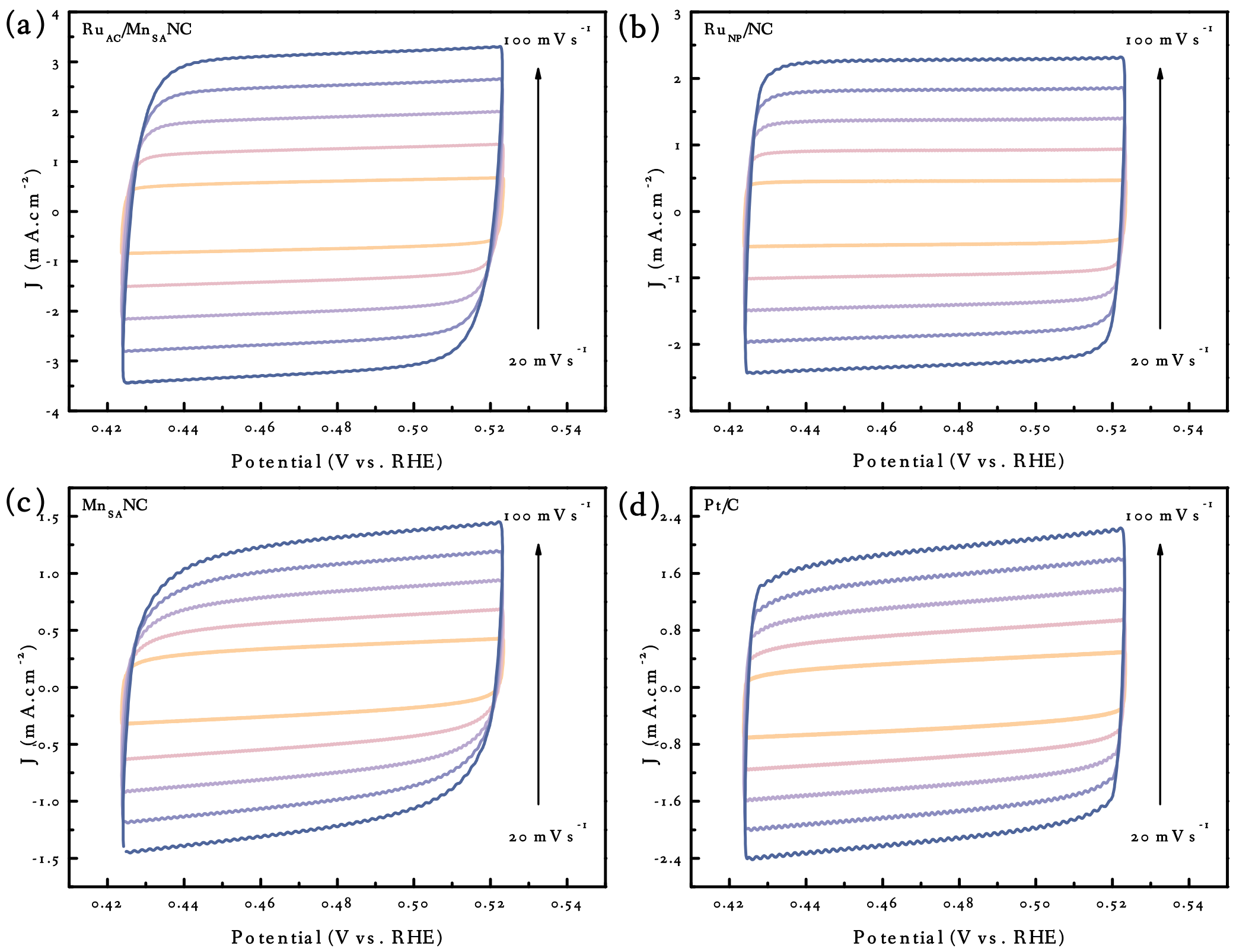

Figure S10. Cyclic voltammograms measured in non-Faradaic region of the voltammogram at different scan rate of a) $Ru_{AC}/Mn_{SA}NC$, b) Ru_{NP}/NC, c) $Mn_{SA}NC$ and d) Pt/C.

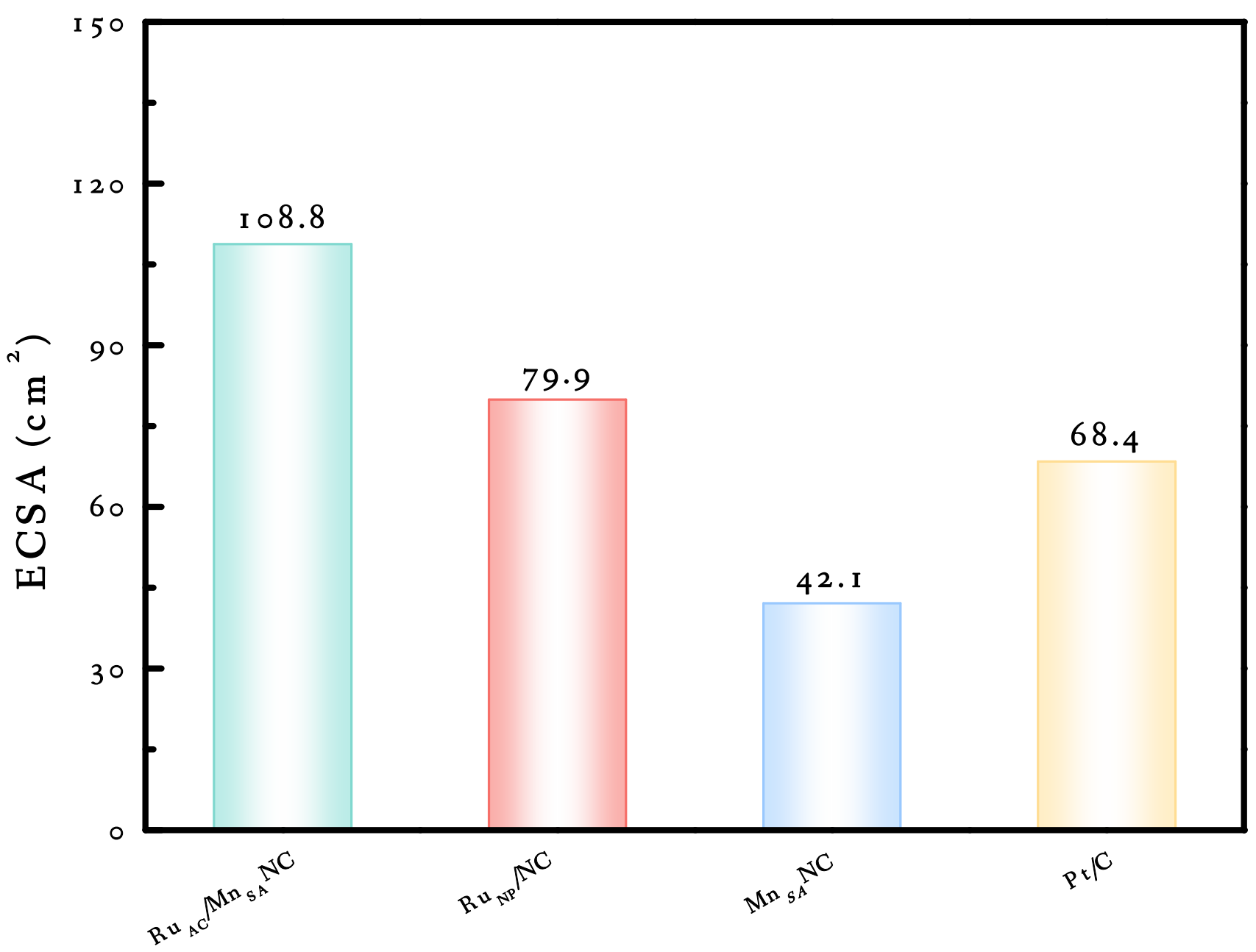

Figure S11. Electrochemical surface area (ECSA) of $Ru_{AC}/Mn_{SA}NC$, Ru_{NP}/NC, $Mn_{SA}NC$ and Pt/C.

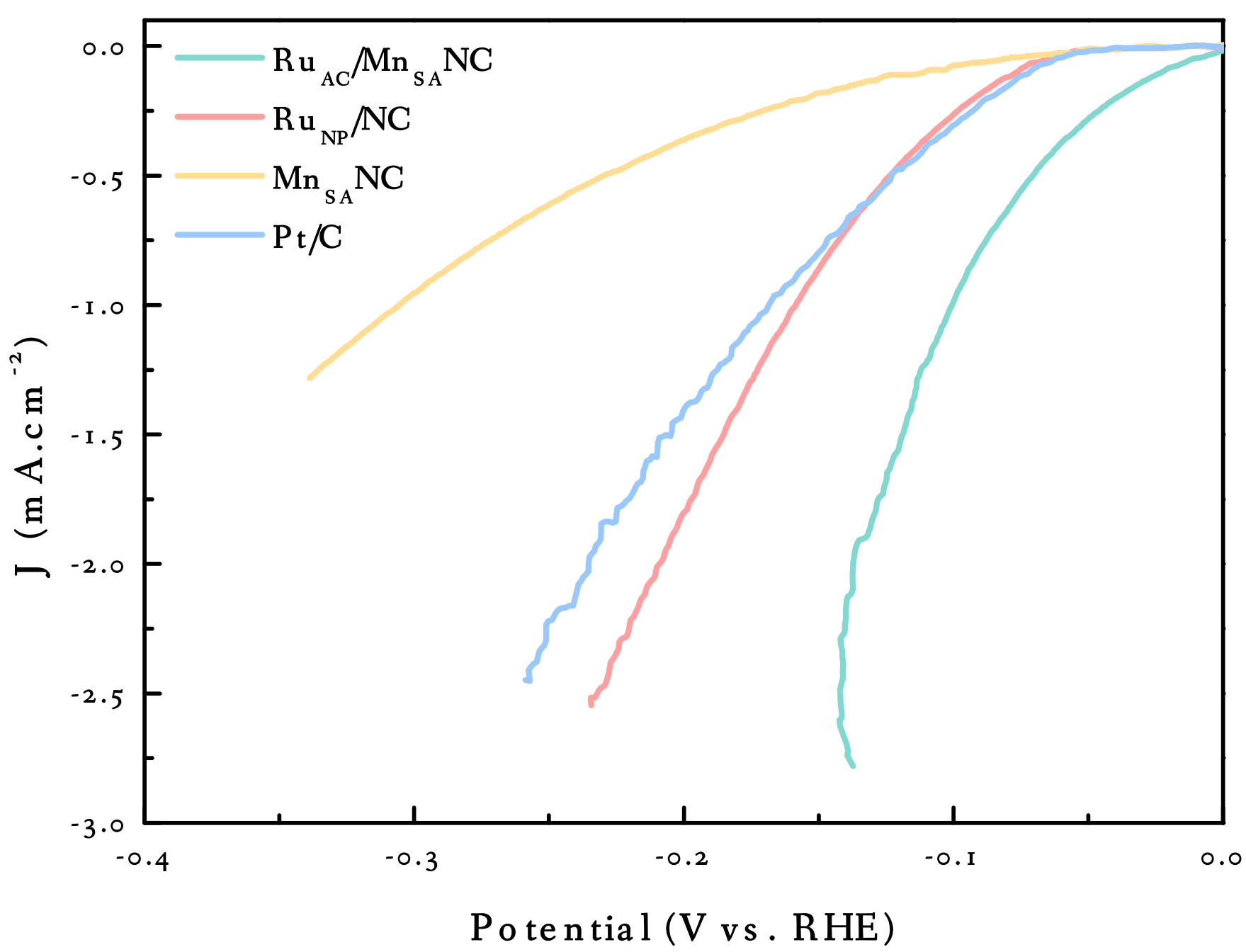

Figure S12. Specific HER activity normalized by the ECSA of the $Ru_{AC}/Mn_{SA}NC$, Ru_{NP}/NC, $Mn_{SA}NC$ and Pt/C.

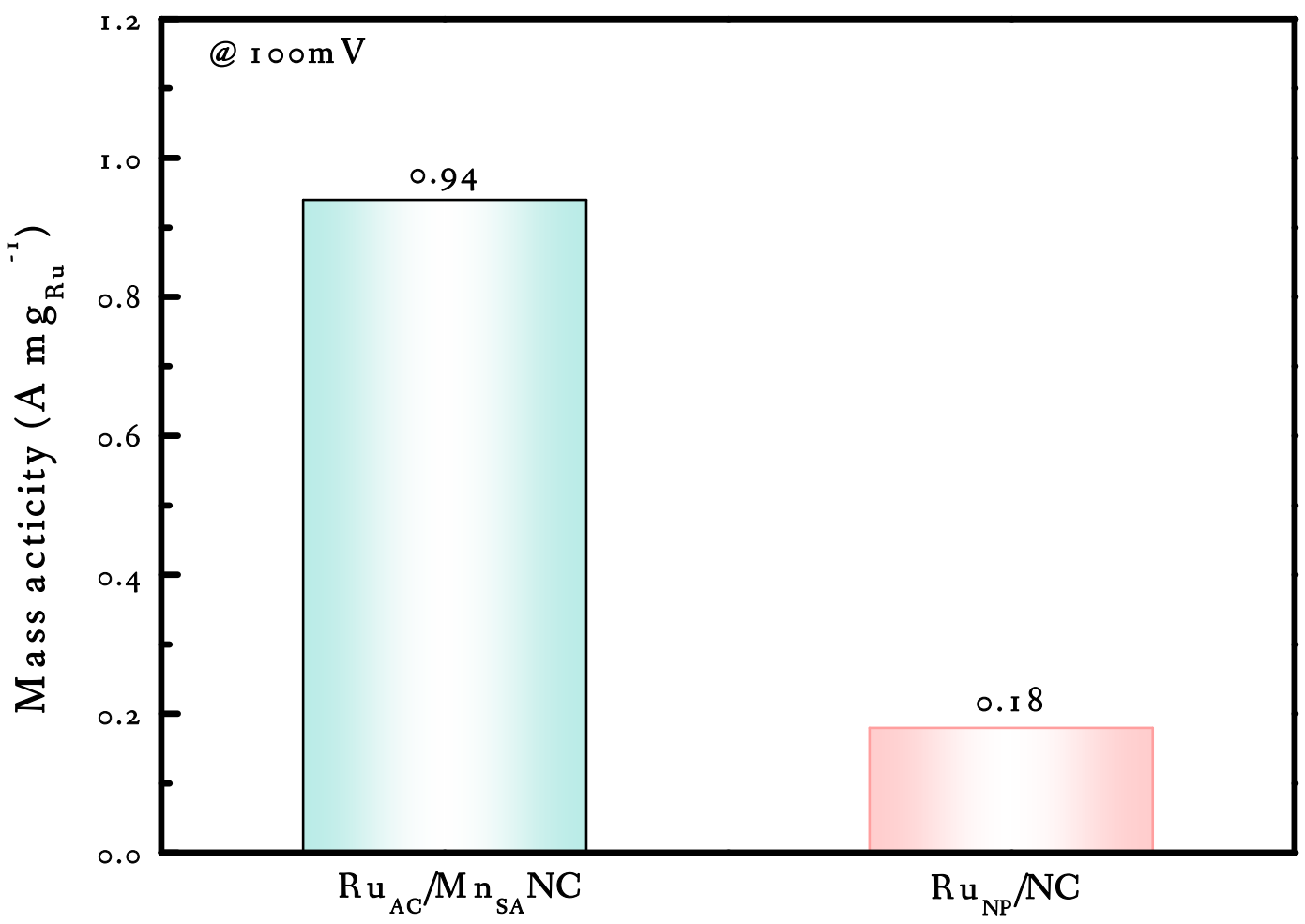

Figure S13. Mass activity of $Ru_{AC}/Mn_{SA}NC$ and Ru_{NP}/NC.

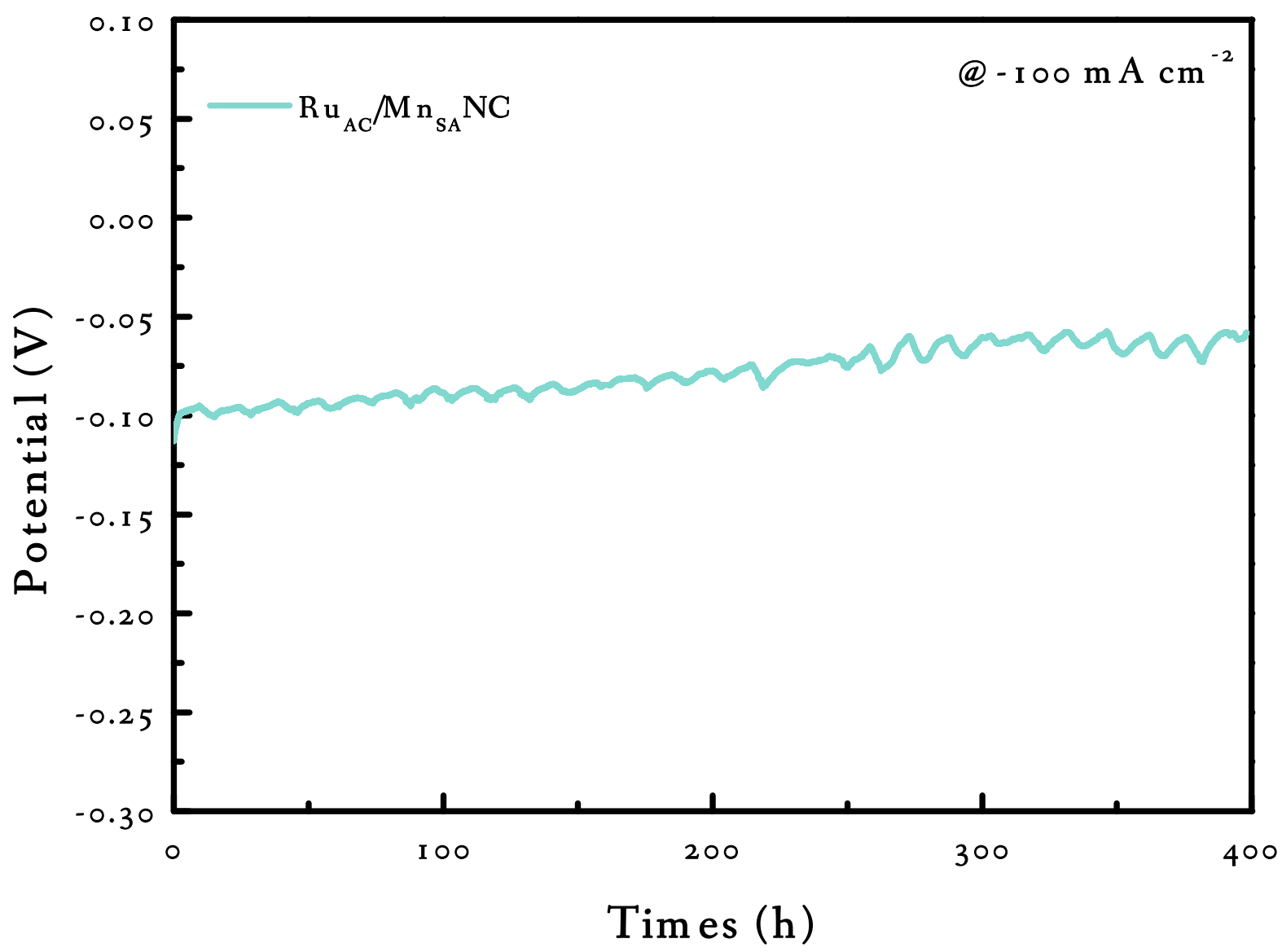

Figure S14. Chronopotentiometry curves of $Ru_{AC}/Mn_{SA}NC$ at -100 mA cm^{-2}.

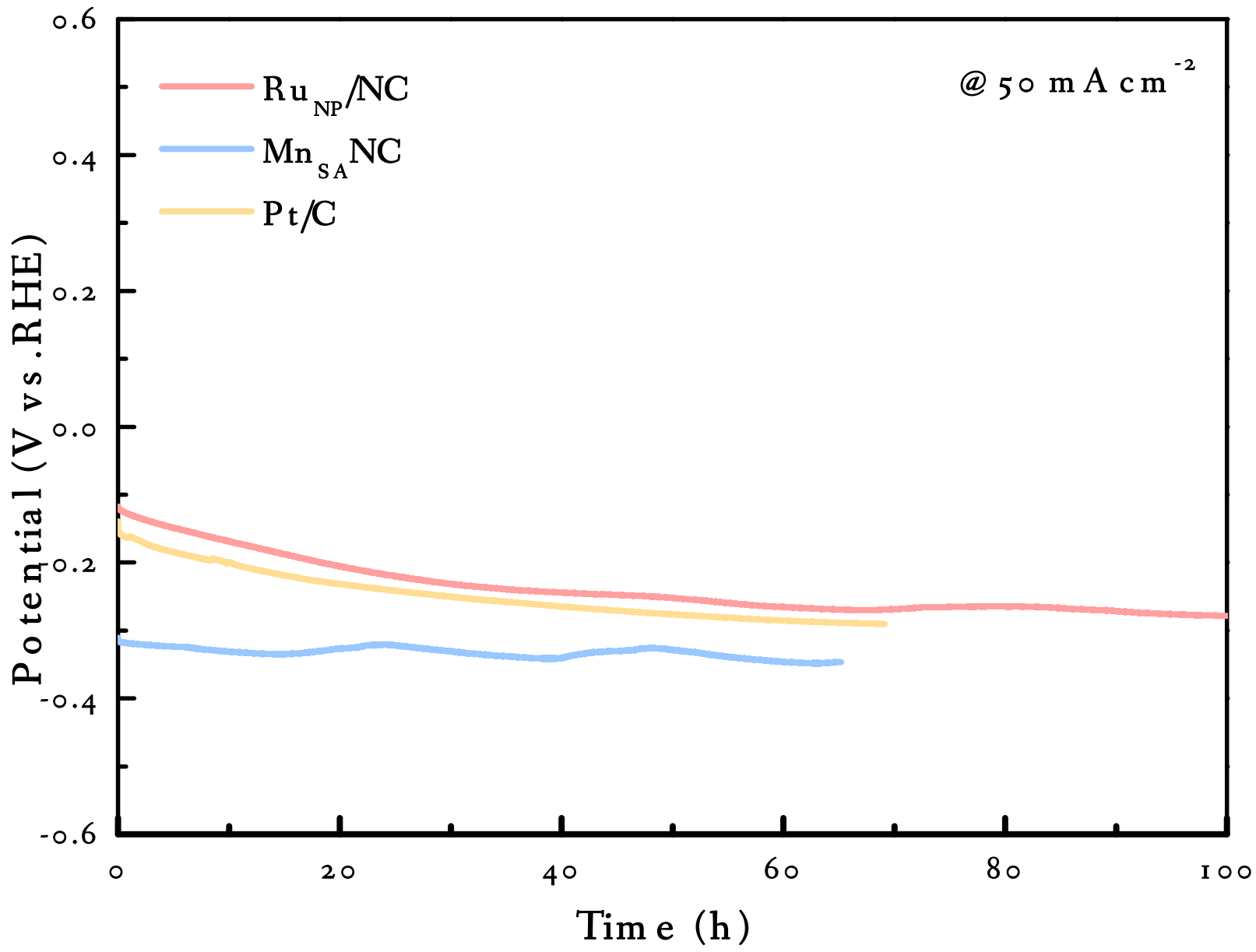

Figure S15. Chronopotentiometry curves of Ru_{NP}/NC, Mn_{SA}NC and Pt/C.

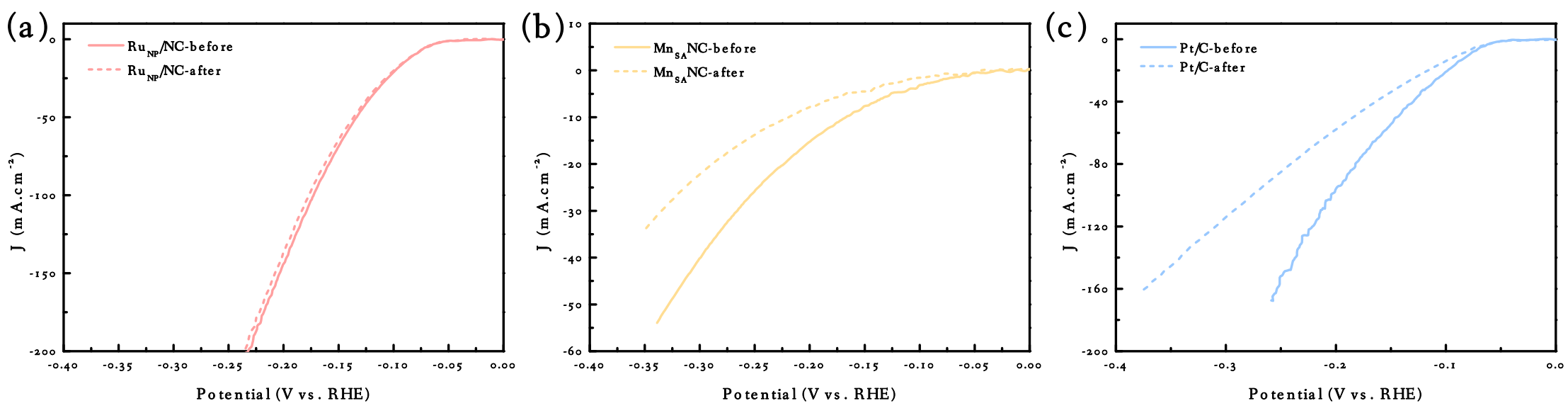

Figure S16. LSV curves of Ru_{NP}/NC, Mn_{SA}NC and Pt/C before and after tests.

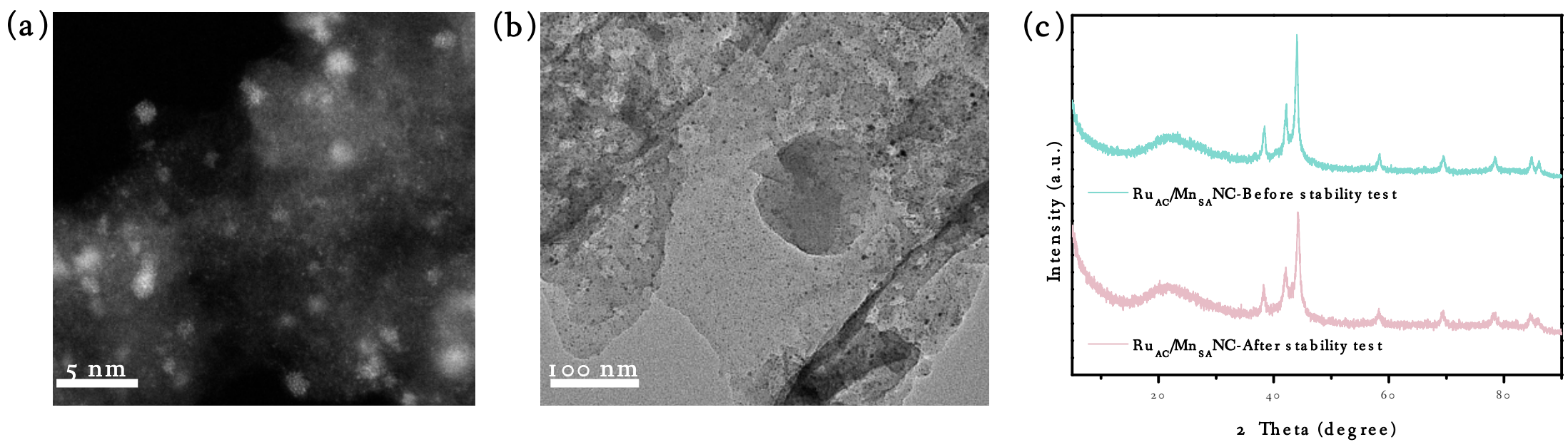

Figure S17. (a) Aberration-corrected HAADF STEM and (b) TME image of $Ru_{AC}/Mn_{SA}NC$ after stability test. (c) XRD pattern of of $Ru_{AC}/Mn_{SA}NC$ before and after stability test.

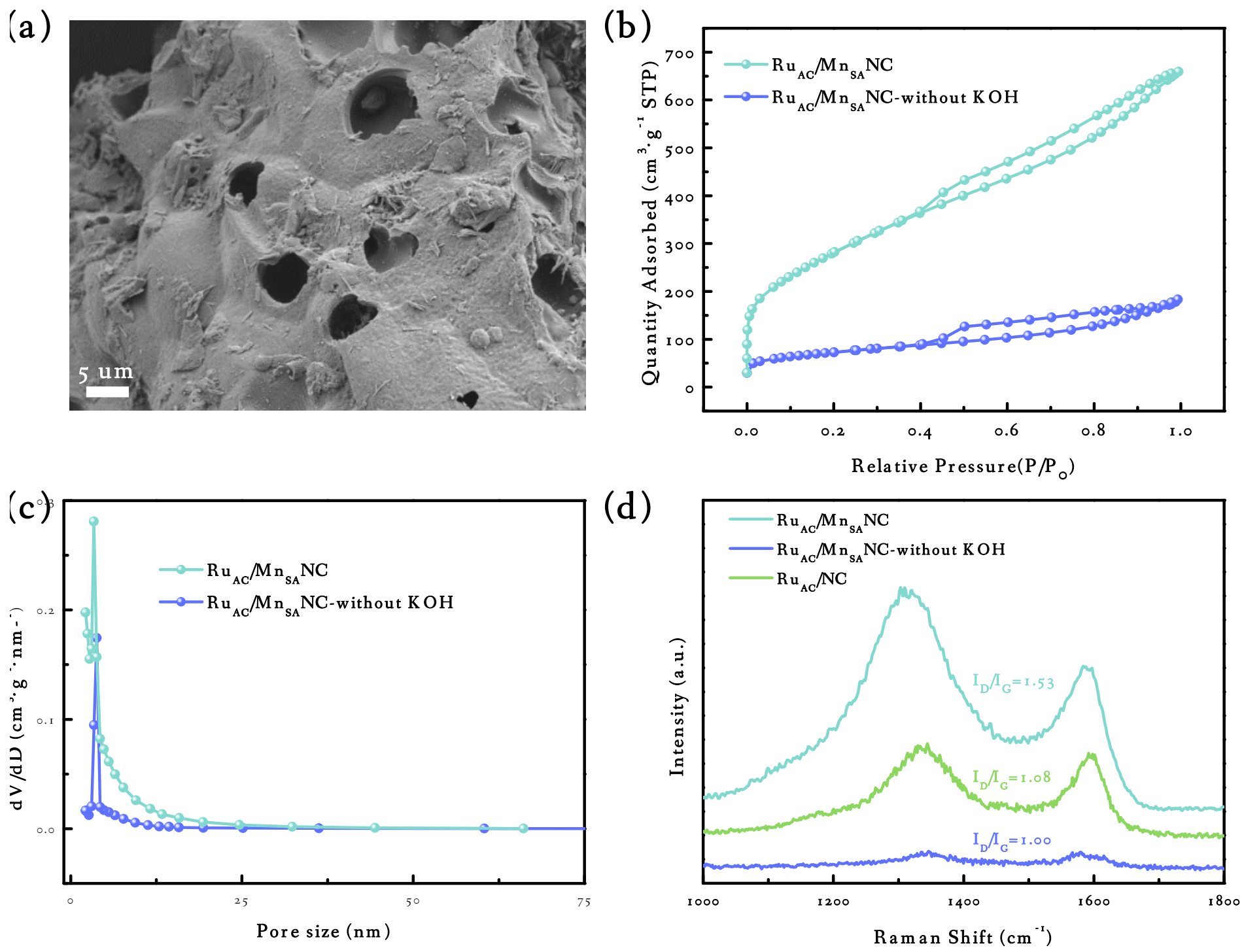

Figure S18. (a) The SEM image of $Ru_{AC}/Mn_{SA}NC$-without KOH. (b,c) N_2 adsorption-desorption isotherms and pore-size distribution curves of $Ru_{AC}/Mn_{SA}NC$ and $Ru_{AC}/Mn_{SA}NC$-without KOH. (d) Raman spectra of $Ru_{AC}/Mn_{SA}NC$, Ru_{AC}/NC and $Ru_{AC}/Mn_{SA}NC$-without KOH.

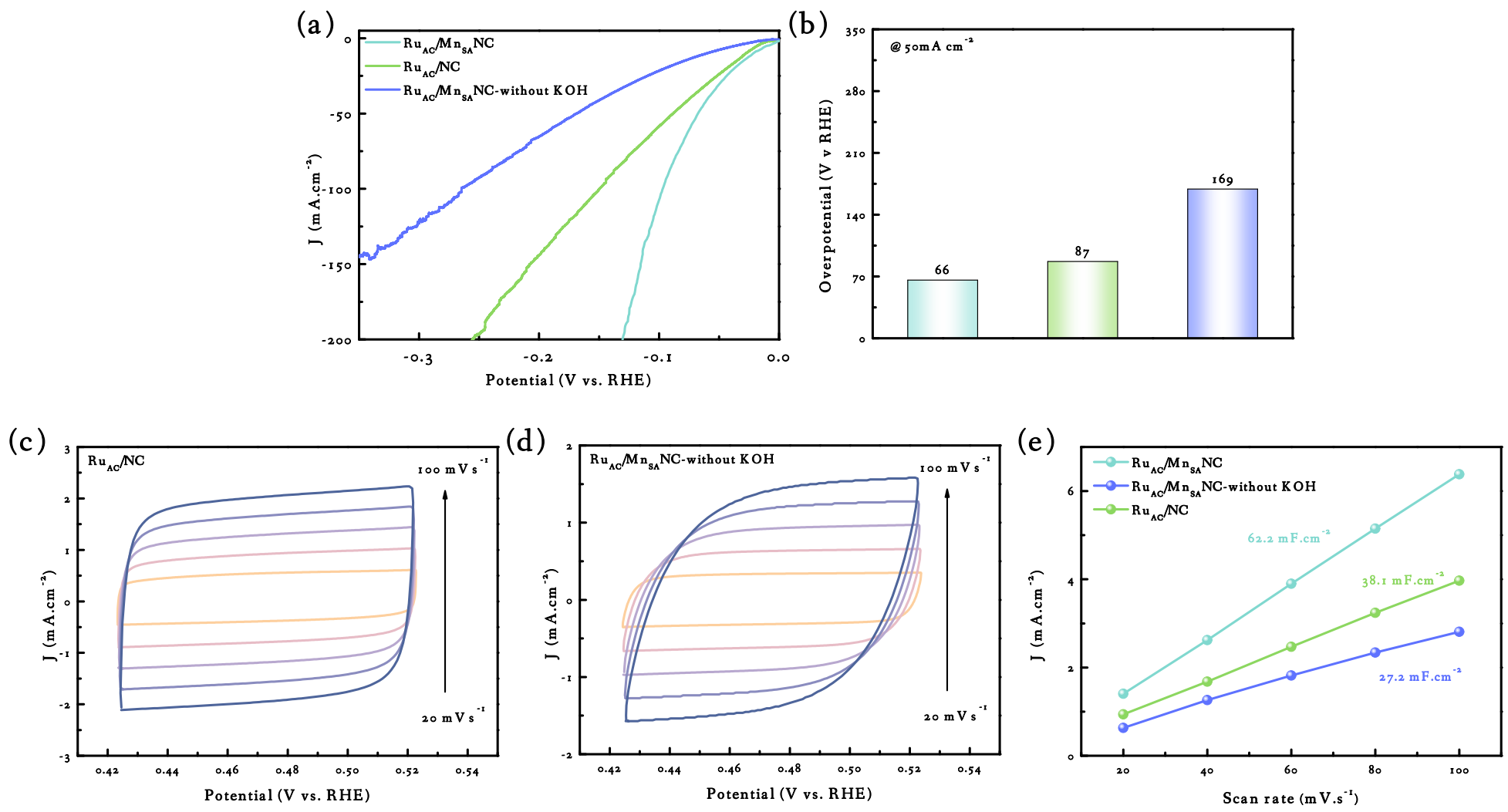

Figure S19. (a) LSV profiles and (b) Overpotentials of $Ru_{AC}/Mn_{SA}NC$, Ru_{AC}/NC and $Ru_{AC}/Mn_{SA}NC$-without KOH. (c) Cyclic voltammograms measured in non-Faradaic region of the voltammogram at different scan rate of (c) Ru_{AC}/NC and (d) $Ru_{AC}/Mn_{SA}NC$-without KOH. (e) Electrochemically active surface area (ECSA) derived from C_{dl} values.

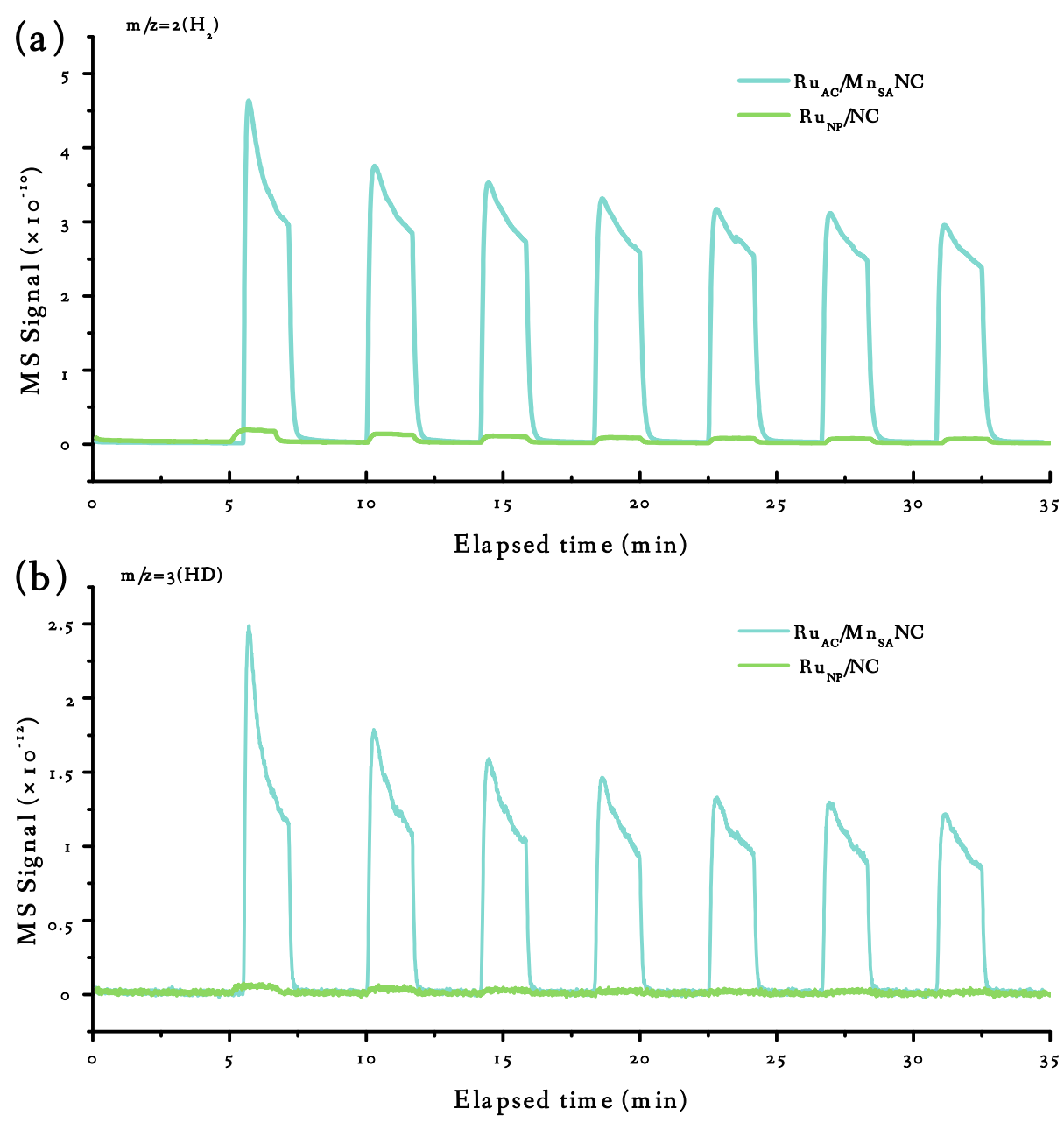

Figure S20. Time-resolved DEMS signals of (a) H_2 (m/z = 2) and (b) HD (m/z = 3) for $Ru_{AC}/Mn_{SA}NC$ and Ru_{NP}/NC measured in H_2O-based electrolyte following D_2O labeling.

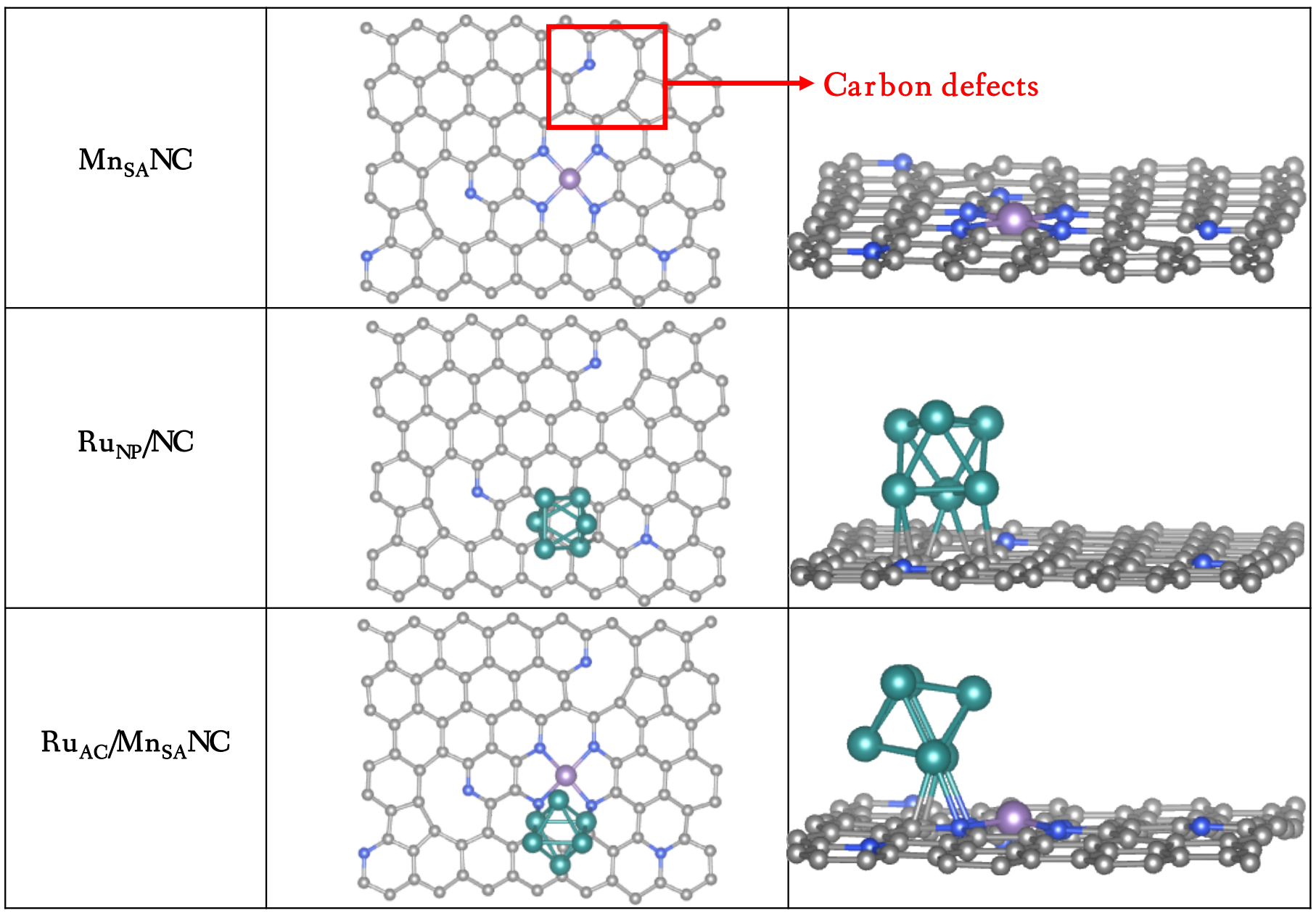

Figure S21. The optimized theoretical models for $Ru_{AC}/Mn_{SA}NC$, Ru_{NP}/NC, and $Mn_{SA}NC$.

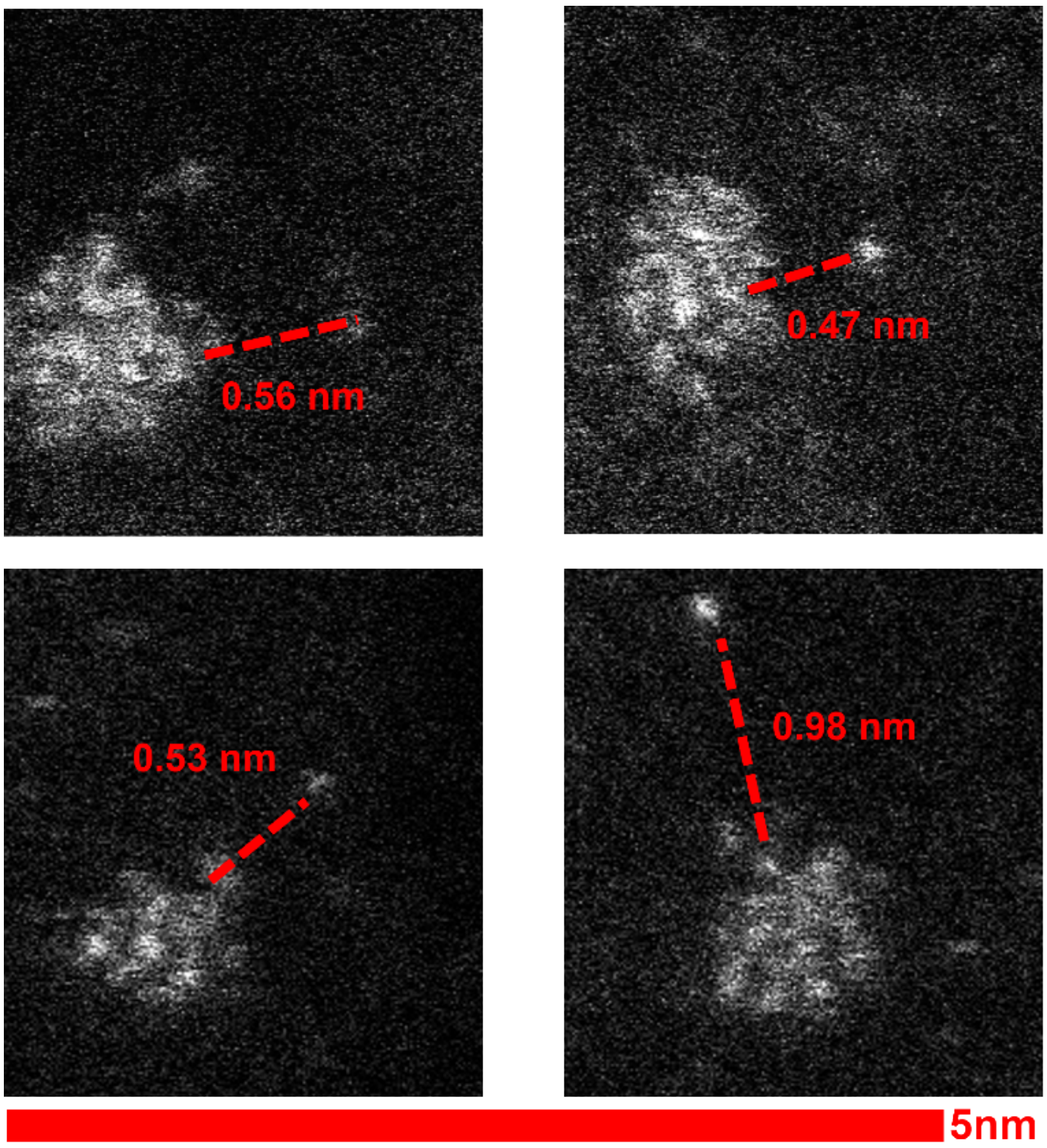

Figure S22. Aberration-corrected HAADF STEM image at different positions.

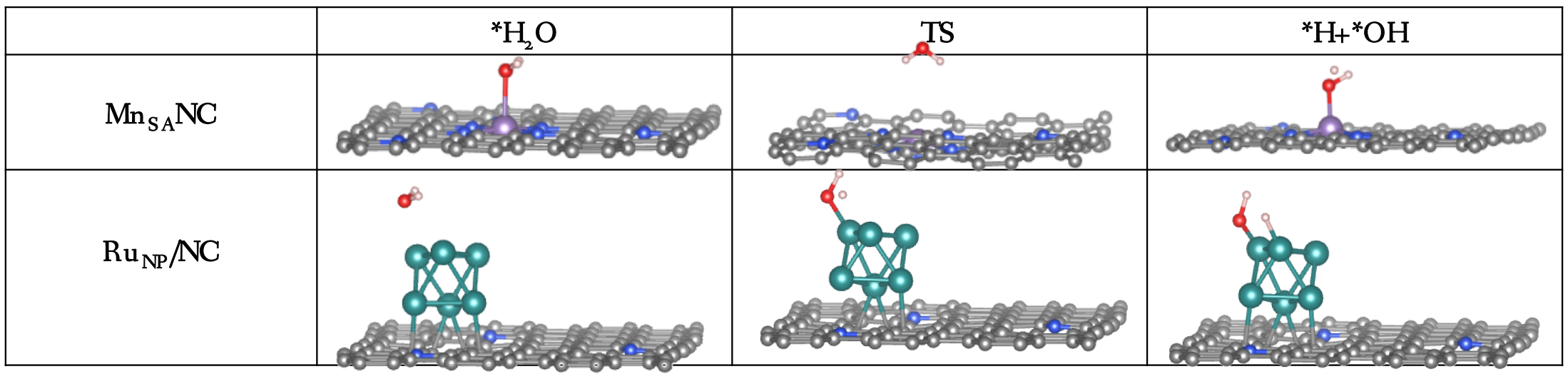

Figure S23. Schematic of the whole HER mechanism on Ru_{NP}/NC and Mn_{SA}NC in the alkaline electrolyte.

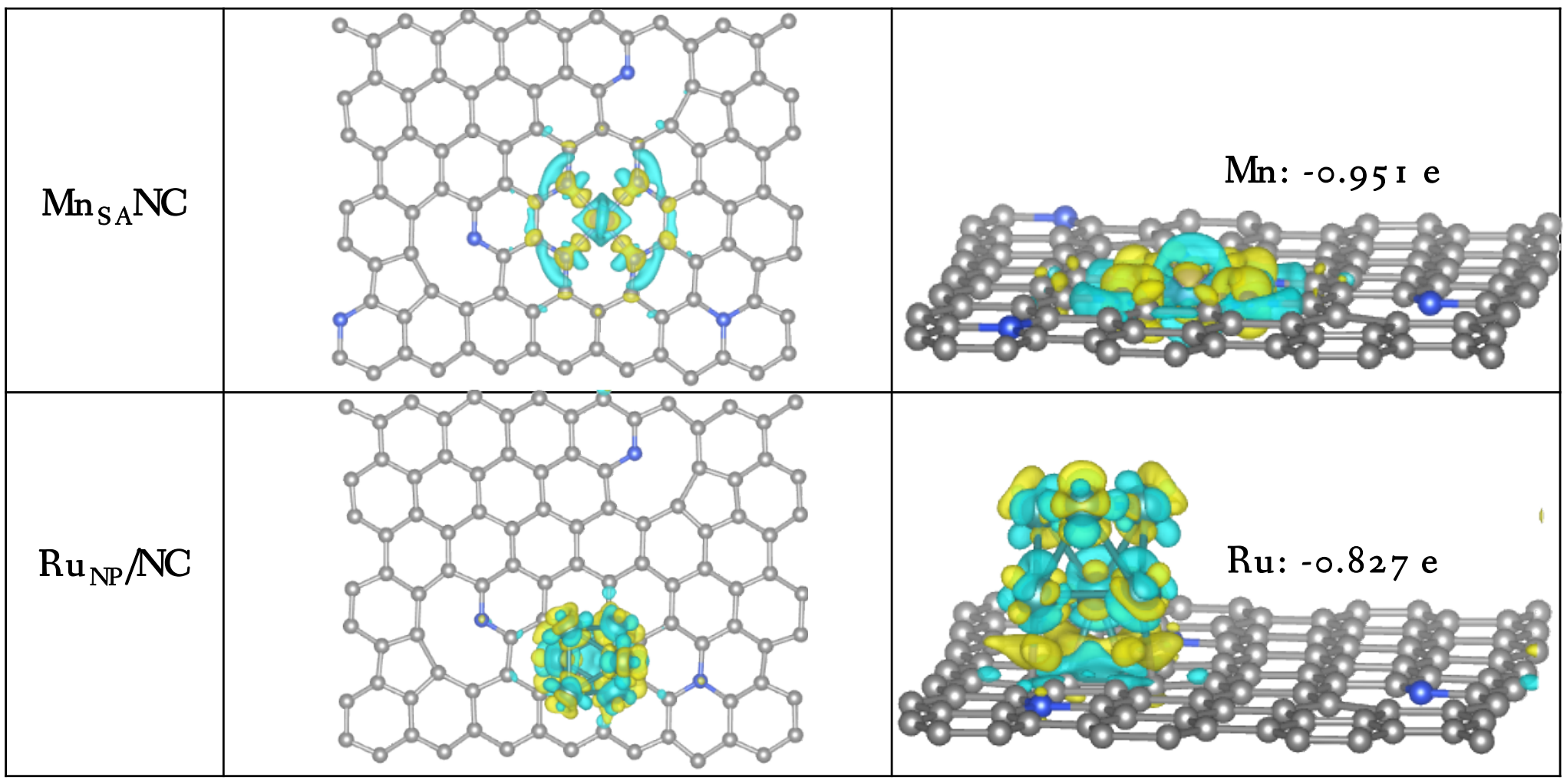

Figure S24. Charge density images of Ru_{NP}/NC and Mn_{SA}NC catalysts.

Table S1 Mass content of before and after stability test determined by ICP.

Catalyst	ICP Loading by at % (Before)		ICP Loading by at % (After)	
	**Ru**	**Mn**	**Ru**	**Mn**
$Ru_{AC}/Mn_{SA}NC$	9.87	1.04	8.79	0.89
Ru_{NP}/NC	10.09	--	7.65	--
$Mn_{SA}NC$	--	1.23	--	0.75

Table S2. EXAFS fitting parameters at the Mn K-edge for $Ru_{AC}/Mn_{SA}NC$.

Sample	Path	CN	R(Å)	σ^2(Å^2)	R-factor
$Ru_{AC}/Mn_{SA}NC$	Mn-N	4.02	2.15	0.0016	0.0054
$Ru_{AC}/Mn_{SA}NC$	Ru-Ru	5.5	2.67	0.0027	0.0047

Table S3. EXAFS fitting parameters at the Ru K-edge for $Ru_{AC}/Mn_{SA}NC$.

Sample	Path	CN	R(Å)	σ^2(Å^2)	R-factor
$Ru_{AC}/Mn_{SA}NC$	Ru-Ru	5.5	2.67	0.0027	0.0047

Table S4 The comparison of HER activities of $Ru_{AC}/Mn_{SA}NC$ with other reported catalysts in 1.0 M KOH.

	**Samples**	**η50 (mV)**	Tafel Slope (mV dec^{-1})	**Reference**
1	$Ru_{AC}/Mn_{SA}NC$	66	24.3	This work
2	Ru/WO_3-V_O	69	35.8	[7]
3	RMNCL	66	66	[8]
4	CoP–Ru	63	32.8	[9]
5	$Ru_{1,n}$-$ZnFe_2O_x$-C	50	22	[10]
6	Ru-NC	50	46	[11]
7	Mo_2C-Ru/C	52	25	[12]
8	Ru/Co_3O_4 NWs	98	69	[13]
9	Pt/Ru NWs	100	26.3	[14]
10	Ru_{SA+AC}/np-Mo	90	32.4	[15]
11	Ru/P-TiO_2	80	28.3	[16]
12	Ru–YNC	75	39	[17]

Reference

[1] Kresse, G.; Furthmüller, J. Efficient Iterative Schemes for Ab Initio Total-Energy Calculations Using

a Plane-Wave Basis Set. Phys. Rev. B 54(1996) 11169-11186.

[2] Perdew, J. P.; Burke, K.; Ernzerhof, M. Generalized Gradient Approximation Made Simple. Phys. Rev. Lett. 77(1996) 3865-3868.

[3] Kresse, G.; Joubert, D. From Ultrasoft Pseudopotentials to the Projector Augmented-Wave Method. Phys. Rev. B 59(1999) 1758-1775.

[4] Blöchl, P. E. Projector Augmented-Wave Method. Phys. Rev. B 50(1994) 17953–17979.

[5] Grimme, S.; Antony, J.; Ehrlich, S.; Krieg, H. J. Chem. Phys. 132(2010) 154104.

[6] H. J. Monkhorst, J. D. Pack, Phys. Rev B. 13(1976) 5188-5192.

[7] J. Cai, W. Zhang, Y. Liu, R. Shen, X. Xie, W. Tian, X. Zhang, J. Ding, Y. Liu, B. Li, Interfacial synergistic effect of Ru nanoparticles embedded onto amorphous/crystalline WO_3 nanorods on boosting the pH-universal hydrogen evolution reaction, Appl. Catal. B 343 (2024) 123502.

[8] W. Ma, Y. Zhang, B. Wang, J. Wang, Y. Dai, L. Hu, X. Lv, J. Dang, Significantly enhanced OER and HER performance of NiCo-LDH and NiCoP under industrial water splitting conditions through Ru and Mn bimetallic co-doping strategy, Chem. Eng. J. 494 (2024) 153212.

[9] X. Wang, J. Zhang, Z. Wang, Z. Lin, S. Shen, W. Zhong, Fabricating Ru single atoms and clusters on CoP for boosted hydrogen evolution reaction, Chin. J. Struct. Chem. 42(4) (2023) 100035.

[10] C. Qian, W. Shao, X. Zhang, X. Mu, X. Gu, M. Yu, L. Ma, S. Liu, S. Mu, Competitive Coordination-Pairing between Ru Clusters and Single-Atoms for Efficient Hydrogen Evolution Reaction in Alkaline Seawater, Small 18(40) (2022) 2204155.

[11] Z. Sun, Q. Guo, J. Liu, H. Liang, J. Bai, S. Ding, Y. Su, Modulating electronic asymmetry in Ru clusters to boost hydrogen evolution reaction, Nano Res. 18(9) (2025) 94907689.

[12] R. Tang, Y. Yang, Y. Zhou, X.-Y. Yu, Rational Design of Heterostructured Ru Cluster-Based Catalyst for pH Universal Hydrogen Evolution Reaction and High-Performance Zn-H_2O Battery, Adv. Funct. Mater. 34(5) (2024) 2301925.

[13] Z. Liu, L. Zeng, J. Yu, L. Yang, J. Zhang, X. Zhang, F. Han, L. Zhao, X. Li, H. Liu, W. Zhou, Charge redistribution of Ru nanoclusters on Co_3O_4 porous nanowire via the oxygen regulation for enhanced hydrogen evolution reaction, Nano Energy 85 (2021) 105940.

[14] J. Yan, R. Wu, G. Jin, L. Jia, G. Feng, X. Tong, The hybrid Pt nanoclusters/Ru nanowires catalysts accelerating alkaline hydrogen evolution reaction, Adv. Powder Mater. 3(5) (2024) 100214.

[15] C. Jian, J. Yuan, T. Wang, L. Zeng, W. Liu, Construction of Ru Single-Atom and Cluster/np-Mo

synergistic active sites with hydrogen spillover for efficient hydrogen evolution reaction, Chem. Eng. J. 510 (2025) 161683.

[16] S. Zhou, H. Jang, Q. Qin, L. Hou, M.G. Kim, S. Liu, X. Liu, J. Cho, Boosting Hydrogen Evolution Reaction by Phase Engineering and Phosphorus Doping on Ru/P-TiO_2, Angew. Chem. Int. Ed. 61(47) (2022) e202212196.

[17] H. Zhang, H. Guo, F. Zhang, J. Zhang, Y. Cheng, Y. Ma, L. Ma, L. Qi, Cascade electrocatalysis via integration of ruthenium clusters and yttrium single atoms for a boosted alkaline hydrogen evolution reaction, Energy Environ. Sci. 18(12) (2025) 6141-6153.
